# The partly parametric and partly nonparametric additive risk model

**DOI:** 10.1007/s10985-021-09535-3

**Published:** 2021-10-26

**Authors:** Nils Lid Hjort, Emil Aas Stoltenberg

**Affiliations:** 1 Department of Mathematics, University of Oslo, Oslo, Norway; 2 BI Norwegian Business School, Oslo, Norway

**Keywords:** Counting processes, Event history, Goodness of fit processes, Linear hazard regression model, Semiparametric

## Abstract

Aalen’s linear hazard rate regression model is a useful and increasingly popular alternative to Cox’ multiplicative hazard rate model. It postulates that an individual has hazard rate function $h(s)=z_1\alpha _1(s)+\cdots +z_r\alpha _r(s)$ in terms of his covariate values $z_1,\ldots ,z_r$. These are typically levels of various hazard factors, and may also be time-dependent. The hazard factor functions $\alpha _j(s)$ are the parameters of the model and are estimated from data. This is traditionally accomplished in a fully nonparametric way. This paper develops methodology for estimating the hazard factor functions when some of them are modelled parametrically while the others are left unspecified. Large-sample results are reached inside this partly parametric, partly nonparametric framework, which also enables us to assess the goodness of fit of the model’s parametric components. In addition, these results are used to pinpoint how much precision is gained, using the parametric-nonparametric model, over the standard nonparametric method. A real-data application is included, along with a brief simulation study.

## Introduction and summary

Suppose individual *i* has observable covariate values $z_{i,1},\ldots ,z_{i,r}$ and that these are thought to influence the probability distribution of his life time $T_i$. The most usual way of modelling this is through Cox’ regression model for the hazard rate $h_i(s)$, which takes this to be of the form $h_0(s)\exp (\beta _1z_{i,1}+\cdots +\beta _rz_{i,r})$ for certain parameters $\beta _1,\ldots ,\beta _r$. Aalen’s linear hazard rate regression model has over the past few decades become a useful and popular alternative. It postulates that individual *i* has hazard rate function

1.1 $$\begin{aligned} h_i(s)=h(s\,|\,z_i) =z_{i,1}\alpha _1(s)+\cdots +z_{i,r}\alpha _r(s) =z_i^\mathrm{t}\alpha (s), \end{aligned}$$

where the $\alpha _j(s)$ functions are unknown. The observed data comprise triples $(t_i,\delta _i,z_i)$, for individuals $i=1,\ldots ,n$, where $\delta _i$ is an indicator for non-censoring. See Aalen (1980, 1989, 1993) and the relevant chapters of the classic monographs Andersen, Borgan, Gill and Keiding (1993, Ch. VII) and Aalen, Borgan and Gjessing (2008, Ch. VI) for general discussion of the (1.1) model, for the most usual estimation methods and their properties, and for applications to various datasets. We comment below on various extensions of and further developments for the basic Aalen model (1.1). The present paper is yet another contribution to this literature, taking some of the regressor functions parametric and the others nonparametric.

One may think of $z_j=z_{i,j}$ as the level of hazard factor no. *j* for the individual, and the $\alpha _j(s)$ function as the associated hazard factor function, or regressor function. Often, the first covariate is the constant 1, and the others are scaled such that zero is the minimum value when the covariate is discrete, or the mean value when the covariate is continuous, in which case one typically also scales the covariate by the inverse of the empirical standard deviation. In such cases equation (1.1) models hazard rate as the common $\alpha _1(s)$ plus excess contributions due to hazard factors $z_2,\ldots ,z_r$. The covariates may also depend upon time as long as they do so in a previsible or predictable fashion; the covariate values $z_i(s)$ at time *s* should be known just prior to time *s*. It suffices that the $z_i(s)$ are left-continuous functions of what has been observed on [0, *s*], i.e., they must not depend on information becoming available after *s*.

Importantly, the Aalen additive model is typically estimated nonparametrically, where there are no further assumptions beyond positivity and continuity of $z_i^\mathrm{t}\alpha (s)$ of (1.1) for all $z_i$ in the support of the distribution of covariates. For the typical application, nonparametric estimates of the cumulative hazard factor functions

1.2 $$\begin{aligned} A_j(t)=\int _0^t\alpha _j(s)\,\mathrm{d}s \quad \mathrm{for\ }j=1,\ldots ,r \end{aligned}$$

are computed and displayed, supplemented with variability estimates. This is used to suggest conclusions about relative influence over time of the different covariate factors. The survival curves for given individuals may also be read off from the modelling here, and if an individual has covariate vector $z=(z_1,\ldots ,z_r)^\mathrm{t}$, not changing over time, the survival curve is

1.3 $$\begin{aligned} S(t\,|\,z)=\exp \{-z^\mathrm{t}A(t)\} =\exp \{-z_1A_1(t)-\cdots -z_rA_r(t)\}. \end{aligned}$$

There has been considerable further research, extending and finessing aspects of the basic Aalen model (1.1)–(1.3), see e.g. Martinussen and Scheike (2007, 2002a, 2002b, 2009b, 2009a). McKeague and Sasieni (1994) studies a version where some of the $\alpha _j(s)$ functions are taken constant, the other taken nonparametric; the present paper extends these ideas and methods further. Stoltenberg (2020) studies the Aalen model in the presence of a cure fraction. Borgan et al. (2007) extend certain features of the model to encompass recurrent event data and to reflect between-subject heterogeneity and missing data. Also of relevance for the present paper, Jullum and Hjort (2017) develop general model selection methods for choosing among parametric and nonparametric candidate models; and Jullum and Hjort (2019) study the possible efficiency gains in specifying a parametric baseline hazard in the Cox regression model.

In applications, the researcher might have firm prior opinions about the functional form of the effect of certain covariates, while being less informed about others. This motivates a framework where some of the hazard factor functions, say the first *p*, are specified parametrically, while the remaining $q=r-p$ continues to be left unspecified, beyond the basic requirement that the (1.1) quantity is nonnegative across all expected covariate values, for all times *s*. Writing $z_{i,(1)}$ for the first *p* components and $z_{i,(2)}$ for the remaining *q* of $z_i$, with a similar block division of $\alpha (s)$ into $\alpha _{(1)}(s,\theta )$ and $\alpha _{(2)}(s)$, the model becomes

1.4 $$\begin{aligned} \begin{aligned} h_i(s)&= \displaystyle z_{i,(1)}^\mathrm{t}\alpha _{(1)}(s,\theta ) +z_{i,(2)}^\mathrm{t}\alpha _{(2)}(s) \\&= \displaystyle \sum _{j=1}^p z_{i,j}\alpha _j(s,\theta ) +\sum _{j=p+1}^{p+q} z_{i,j}\alpha _j(s) \end{aligned} \end{aligned}$$

for $i=1,\ldots ,n$. Here $\theta$ is the collection of parameters used to describe the first *p* hazard factor functions, which would typically take the form $\alpha _j(s,\theta _j)$ for $j=1,\ldots ,p$.

The covariates in (1.4) are not dependent on time. As discussed in relation to the Aalen model of (1.1), an extension to time-varying covariates requires only minor modifications to the theory related to predictability and linear independence of the the covariates at all time points. To ease the presentation we stick to covariates that are constant in time.

Our quest is two-fold. We aim first at developing sound estimation methods for the unknowns of the (1.4) model, along with large-sample theory describing the behaviour of these estimators. Secondly, accompanying goodness-of-fit measures will be constructed, to assess the adequacy of the parametric components. To reach these goals our paper proceeds as follows.

We start in Sect. 2 by presenting the natural methods and results for the purely nonparametric and the purely parametric versions of model (1.4), before going on to our favoured estimation strategies for the cases with both parametric and nonparametric components in Sect. 3. In Sect. 4 we derive the required large-sample normality results, for both the parametric and nonparametric parts, enabling statistical inference. A special case of the class of methods we propose is asymptotically optimal; the details concerning such statements have independent interest, and are summarised in Appendix A. Part of the benefit of using parametric rather than nonparametric components in the (1.1) model is that it leads to better precision, again for both the parametric and nonparametric components; this is assessed and illustrated in Appendix B.

Then in Sect. 5 we construct goodness-of-fit monitoring processes, which in particular lead to classes of chi-squared tests. In Sect. 6 the finite-sample behaviour of our estimation and inference methods is illustrated through a simulation study. We also present an empirical application, related to $n=312$ primary biliary cirrhosis patients in a double-blind randomised study, comparing our methods to those associated with the fully nonparametric Aalen estimator. These applications illustrate the usefulness of the our methods, and showcase the gains in efficiency that are achieved by going partly parametric partly nonparametric, as opposed to fully nonparametric. Our article ends with a list of remarks, some pointing to further research work, in Sect. 7.

## The fully nonparametric and fully parametric cases

Here we establish some notation and briefly describe the estimators $\widetilde{A}_1,\ldots ,\widetilde{A}_r$ in typical use for the full nonparametric model, in Sects. 2.1–2.2. These will be the basis for fitting the parametric and nonparametric components in later sections. We also go through the natural estimation methods for the special case of (1.4) where all components are specified parametrically, in Sect. 2.3.

We first go through and comment on certain assumptions of convenience, which will be taken to hold throughout our article.

### Assumptions 1

(1) *Ergodicity:* All averages $$n^{-1}\sum _{i=1}^n\phi (z_i)$$ converge to appropriate limits as *n* grows. These limits may be interpreted as means with respect to the covariate distribution. This assumption facilitates the mathematical development and makes it easier to give precise statements about e.g. limit distributions of estimators. The large-sample theory is, however, developed *conditionally* on the observed covariate values, so all randomness lies in $$(T_i,\delta _i)$$ given these. (2) *Finite time window:* Individuals are followed over a fixed finite time interval, say $$[0,\tau ]$$. This is not a restriction in practice. Most results may be extended to the case of $$\tau =\infty $$, under appropriate assumptions on the censoring mechanism. We shall be content to work with the finite time horizon, with which the martingale limit theory works more smoothly and with fewer technicalities. (3) *Independent censoring and finite variances:* The censoring mechanism involved, leading to data $$(t_i,\delta _i)$$, are not related to the survival mechanism generating the hazard rates. Furthermore, the $$r\times r$$ matrix function $$n^{-1}\sum _{i=1}^nI(T_i\ge s)z_iz_i^\mathrm{t}$$ tends in probability to a matrix with full rank *r*, for each $$s\in [0,\tau ]$$. This means in particular that the censoring distribution does not have a support strictly smaller than $$[0,\tau ]$$, and also that enough linearly independent covariate vectors $$z_i$$ are present in the risk set at time *s*, with increasing *n*. (4) *Smooth parametric components:* The $$\alpha _j(s,\theta )$$ of (1.4) are smooth in $$\theta $$, with continuous first order derivatives $$\alpha _j^*(s,\theta )$$ and second order derivatives $$\alpha _j^{**}(s,\theta )$$, for $$\theta $$ in a neighbourhood around the true parameter $$\theta _0$$. $$\square $$

### The general integrated weighted least squares estimators

The data consist of triples $(t_i,\delta _i,z_i)$ for each of *n* individuals, where $t_i$ is the life-time, possibly right-censored, $\delta _i$ an indicator for non-censoring, and $z_i$ the *r*-dimensional covariate vector, as above. Let $N_i(t)=I\{t_i\le t,\delta _i=1\}$ and $Y_i(t)=I\{t_i\ge t\}$ be the counting process and at risk indicator for individual *i*, and introduce the martingale $M_i(t)=N_i(t)-\int _0^tY_i(s)z_i^\mathrm{t}\alpha (s)\,\mathrm{d}s$. Then

2.1 $$\begin{aligned} \sum _{i=1}^nw_i(s)z_i\,\mathrm{d}N_i(s)=\sum _{i=1}^nY_i(s)w_i(s)z_iz_i^\mathrm{t}\,\alpha (s)\,\mathrm{d}s +\sum _{i=1}^nw_i(s)z_i\,\mathrm{d}M_i(s), \end{aligned}$$

the second term here being martingale noise with mean zero. Here we have allowed certain weight functions $w_i(s)$ to be used. They are taken to be pre-visible functions (their values at time *s* are known at time $s-$), and the most often used choice is that of $w_i(s)=1$. Equation (2.1) is the motivation behind

2.2 $$\begin{aligned} \mathrm{d}\widetilde{A}(s)= & {} G_n(s)^{-1} n^{-1}\sum _{i=1}^nw_i(s)z_i\,\mathrm{d}N_i(s), \nonumber \\ \mathrm{where}\quad G_n(s)= & {} n^{-1}\sum _{i=1}^nY_i(s)w_i(s)z_iz_i^\mathrm{t}, \end{aligned}$$

with accompanying cumulatives $\widetilde{A}_j(t)=\int _0^t\,\mathrm{d}\widetilde{A}_j(s)$ for $j=1,\ldots ,r$. It is assumed that at least *r* of the $z_i$ at risk at time *s* are linearly independent, so that $G_n(s)$ has full rank.

These estimators have well-studied properties, see Aalen, Borgan and Gjessing (2008, Ch. VI). In particular, large-sample results are available via the calculus of counting processes and martingales. We review briefly here results, and introduce notation which will be needed in the development to follow. Consider

2.3 $$\begin{aligned} U_n(t)=n^{-1/2}\sum _{i=1}^n\int _0^t w_i(s)z_i\,\mathrm{d}M_i(s), \end{aligned}$$

which is a martingale with variance process $H_n(t)=n^{-1}\sum _{i=1}^n\int _0^t Y_i(s)w_i(s)^2z_iz_i^\mathrm{t}\,z_i^\mathrm{t}\alpha (s)\,\mathrm{d}s$. It follows from the regularity conditions described in Assumptions 1 that there are well-defined limits in probability,

$$\begin{aligned} G_n(t)\rightarrow _\mathrm{pr}G(t) \quad \mathrm{and} \quad H_n(t)\rightarrow _\mathrm{pr}H(t), \end{aligned}$$

as *n* increases, where *G* and *H* are full-rank $r\times r$ matrix functions. One finds

2.4 $$\begin{aligned} \sqrt{n}\{\mathrm{d}\widetilde{A}(s)-\mathrm{d}A(s)\} =G_n(s)^{-1}\,\mathrm{d}U_n(s)\rightarrow _d G(s)^{-1}\,\mathrm{d}U(s), \end{aligned}$$

where *U* is a Gaußian martingale with variance level $\mathrm{Var}\,\mathrm{d}U(s)=\mathrm{d}H(s)$. In particular, $\sqrt{n}\{\widetilde{A}(t)-A(t)\}\rightarrow _d\int _0^t G(s)^{-1}\,\mathrm{d}U(s)$, which has variance $\int _0^t G(s)^{-1}\,\mathrm{d}H(s)\,G(s)^{-1}$. This limiting variance may be estimated from data as $\int _0^t G_n(s)^{-1}\,\mathrm{d}\widehat{H}_n(s)\,G_n(s)^{-1}$. There are a couple of options for estimating $\mathrm{d}H(s)$ consistently, including

$$\begin{aligned} \mathrm{d}\widehat{H}_n(s) \!=\!n^{-1}\sum _{i=1}^nY_i(s)w_i(s)^2z_iz_i^\mathrm{t}\,z_i^\mathrm{t}\mathrm{d}\widetilde{A}(s) \mathrm{\ \ and\ \ } \mathrm{d}\widehat{H}(s)\!=\!n^{-1}\sum _{i=1}^nw_i(s)^2z_iz_i^\mathrm{t}\,\mathrm{d}N_i(s). \end{aligned}$$

In our empirical work we have used the second option.

### Optimal nonparametric estimation

One may show, e.g. exploiting a parallel to the theory of weighted least squares, that the theoretically optimal weights, minimising $G_n(s)^{-1}\mathrm{d}H_n(s)G_n(s)^{-1}$, are

2.5 $$\begin{aligned} w_i^0(s)=1/\{z_i^\mathrm{t}\alpha (s)\} \quad \mathrm{for\ }i=1,\ldots ,n. \end{aligned}$$

The resulting minimum variance corresponds to $F_n(s)^{-1}\,\mathrm{d}s$, where

2.6 $$\begin{aligned} F_n(s)=n^{-1}\sum _{i=1}^nY_i(s){z_iz_i^\mathrm{t}\over z_i^\mathrm{t}\alpha (s)}. \end{aligned}$$

In practice one needs to estimate these, say with $\widetilde{w}_i(s)=1/ \{z_i^\mathrm{t}\widetilde{\alpha }(s)\}$, leading to

$$\begin{aligned} \breve{A}(t)=\int _0^t \Bigl \{n^{-1}\sum _{i=1}^nY_i(s)\widetilde{w}_i(s)z_iz_i^\mathrm{t}\Bigr \}^{-1} n^{-1}\sum _{i=1}^n\widetilde{w}_i(z)z_i\,\mathrm{d}N_i(s). \end{aligned}$$

One may show that $\sqrt{n}(\breve{A}-A)$, with estimated optimal weights, has the same limit distribution $\int _0^. F(s)^{-1}\,\mathrm{d}U(s)$ as has $\sqrt{n}(\widetilde{A}-A)$ with optimal weights, provided the $\widetilde{\alpha }(s)$ estimator satisfies certain uniform consistency conditions, see Huffer and McKeague (1991). Candidates for $\widetilde{\alpha }(s)$ include kernel smoothing of the plain Aalen estimators, which use $w_i(s)=1$, and local linear likelihood smoothing. The limit distribution variance for this optimal $\breve{A}$ estimator is $\int _0^tF(s)^{-1}\,\mathrm{d}s$, which is the minimum over all $\int _0^tG(s)\,\mathrm{d}H(s)\,G(s)^{-1}$. Here *F*(*s*) is the limit in probability of $F_n(s)$ of (2.6), assumed to exist.

While $F(s)^{-1}\,\mathrm{d}s$ may be somewhat smaller in size than the most often used $G(s)^{-1}\mathrm{d}H(s)G(s)^{-1}$, with weights $w_i(s)=1$, there are additional variability contributions associated with this estimator, which therefore is not automatically better than the Aalen ones for finite *n*. Our default choice, for empirical work, is therefore to use the ‘plain weights’ $w_i(s)=1$ in (2.2).

### The fully parametric model

Consider now the fully parametric model where $\alpha _j(s)=\alpha _j(s,\theta )$ for $j=1,\ldots ,r$. We study the maximum likelihood estimator $\widehat{\theta }$, maximising the log-likelihood, which may be written

$$\begin{aligned} \ell _n(\theta )=\sum _{i=1}^n\int _0^\tau \bigl [\log \{z_i^\mathrm{t}\alpha (s,\theta )\}\,\mathrm{d}N_i(s) -Y_i(s)z_i^\mathrm{t}\alpha (s,\theta )\,\mathrm{d}s\bigr ]. \end{aligned}$$

Here $\tau$ is an upper bound for the period of observation, assumed finite, see Assumptions 1. Let $\alpha ^*(s,\theta )=\partial \alpha (s,\theta )/\partial \theta$ be the $r\times m$ matrix of partial derivatives $\partial \alpha _j(s,\theta )/\partial \theta _k$, where *m* is the length of the parameter vector $\theta$. Assuming the model holds, with $\theta _0$ the true parameter value, let $\Omega _0=\int _0^\tau \alpha ^*(s,\theta _0)^\mathrm{t}F(s) \alpha ^*(s,\theta _0)\,\mathrm{d}s$, with *F*(*s*) the limit in probability of $F_n(s)$ of (2.6). We then have the following.

#### Proposition 2.1

Under standard regularity conditions, including those described in Assumptions 1, and supposing the model holds for a true parameter $\theta _0$, an inner point of the parameter space, $\sqrt{n}(\widehat{\theta }-\theta _0)$ tends to $\mathrm{N}_m(0,\Omega _0^{-1})$ in distribution.

#### Proof

The proof follows the lines of Borgan (1984) and Hjort (1986, 1992). We need the first and second derivatives of $$z_i^\mathrm{t}\alpha (s,\theta )$$, and write these respectively as $$\alpha ^*(s,\theta )^\mathrm{t}z_i$$, of dimension $$1\times m$$, and $$\sum _{j=1}^rz_{i,j} \alpha ^{**}_j(s,\theta )$$, where $$\alpha ^{**}_j(s,\theta )$$ is the $$m\times m$$ matrix of second order derivatives of $$\alpha _j(s,\theta )$$. The first derivative of $$\ell _n$$ is

$$\begin{aligned} u_n(\theta ) =\sum _{i=1}^n\int _0^\tau \Bigl \{{\alpha ^*(s,\theta )^\mathrm{t}z_i \over \alpha (s,\theta )^\mathrm{t}z_i}\,\mathrm{d}N_i(s) -Y_i(s)\alpha ^*(s,\theta )^\mathrm{t}z_i\,\mathrm{d}s\Bigr \}. \end{aligned}$$

Using the martingales $M_i(t)=N_i(t)-\int _0^t Y_i(s)\alpha (s,\theta _0)^\mathrm{t}z_i\,\mathrm{d}s$ we see that

$$\begin{aligned} n^{-1/2}u_n(\theta _0)=n^{-1/2}\sum _{i=1}^n\int _0^\tau {\alpha ^*(s,\theta _0)^\mathrm{t}z_i\over \alpha (s,\theta _0)^\mathrm{t}z_i} \,\mathrm{d}M_i(s), \end{aligned}$$

which is a martingale, evaluated at $\tau$, with variance process

$$\begin{aligned} J_n= & {} n^{-1}\sum _{i=1}^n\int _0^\tau \Bigl ({\alpha ^*(s,\theta _0)^\mathrm{t}z_i\over \alpha (s,\theta _0)^\mathrm{t}z_i}\Bigr ) \Bigl ({\alpha ^*(s,\theta _0)^\mathrm{t}z_i\over \alpha (s,\theta _0)^\mathrm{t}z_i}\Bigr )^\mathrm{t}Y_i(s)\alpha (s,\theta _0)^\mathrm{t}z_i\,\mathrm{d}s \\= & {} \int _0^\tau \alpha ^*(s,\theta _0)^\mathrm{t}F_n(s)\alpha ^*(s,\theta _0)\,\mathrm{d}s. \end{aligned}$$

It follows that $n^{-1/2}u_n(\theta _0)$ tends to a $\mathrm{N}_m(0,\Omega _0)$ random variable, under model conditions.

We next need to work with the second order derivative $i_n(\theta )$ of $\ell _n$, to show that $-n^{-1}i_n(\theta ) = J_n + o_{\mathrm{pr}}(1)$ at the model. We find

$$\begin{aligned} i_n(\theta )= & {} \sum _{i=1}^n\int _0^\tau \Bigl [{\sum _{j=1}^r z_{i,j} \alpha ^{**}_j(s,\theta ) \alpha (s,\theta )^\mathrm{t}z_i -\{\alpha ^*(s,\theta )^\mathrm{t}z_i\}^2 \over \{\alpha (s,\theta )^\mathrm{t}z_i\}^2}\,\mathrm{d}N_i(s) \\&\qquad \quad -Y_i(s)\sum _{j=1}^r z_{i,j} \alpha ^{**}_j(s,\theta )\,\mathrm{d}s\Bigr ]. \end{aligned}$$

Using the martingales again, and simplifying, shows that

$$\begin{aligned} -n^{-1}i_n(\theta )= & {} n^{-1}\sum _{i=1}^n\int _0^\tau {((\alpha ^*)^\mathrm{t}z_i)^2\over \alpha ^\mathrm{t}z_i}Y_i\,\mathrm{d}s\\&+\, n^{-1}\sum _{i=1}^n\int _0^\tau \Bigl [{((\alpha ^*)^\mathrm{t}z_i)^2\over (\alpha ^\mathrm{t}z_i)^2} -{\sum _{j=1}^r z_{i,j} \alpha ^{**}_j\over \alpha ^\mathrm{t}z_i}\Bigr ]\,\mathrm{d}M_i(s). \end{aligned}$$

At the true value $\theta _0$, the first term is equal to $\int _0^\tau (\alpha ^*)^\mathrm{t}F_n\alpha ^*\,\mathrm{d}s=J_n$, while the second goes to zero in probability, by an application of Lenglart’s inequality, see e.g. Andersen et al. (1993, p. 86). Some further analysis, similar in nature to material in Hjort (1992, Sections 2–3), leads in the end to $\sqrt{n}(\widehat{\theta }-\theta _0)$ being at most $o_\mathrm{pr}(1)$ away from $J_n^{-1}n^{-1/2}u_n(\theta _0)$, which has the limiting $\mathrm{N}_m(0,\Omega _0^{-1})$ distribution. $\square$

## Estimation in the parametric and nonparametric model

In this section we describe estimation methods for the parametric-nonparametric model (1.4). These involve a Step (a) for estimating the parametric parts, the $A_{(1)}(t,\theta )$, with these also being used in a Step (b) for estimating the nonparametric parts. In particular, our estimators for these $A_{(2)}(t)$ utilise the parametric structure for $A_{(1)}(t,\theta )$, and are not identical to the direct Aalen estimators $\widetilde{A}_{(2)}(t)$; the point is to utilise the parametric knowledge, for increased precision.

### Estimating the parametric part

Our preferred version of Step (a) is as follows. It is desirable to find values of $\theta$ which makes the integrated, weighted quadratic form

$$\begin{aligned} \int _0^\tau \{\alpha _{(1)}(s,\theta )-\alpha _{(1)}(s)\}^\mathrm{t}V_n(s)\{\alpha _{(1)}(s,\theta )-\alpha _{(1)}(s)\}\,\mathrm{d}s \end{aligned}$$

as small as possible. Here $\tau$ is an upper time point, which could be chosen by convenience for the application at hand, while the $V_n(s)$ is a full-rank symmetric $p\times p$ matrix weight function. This minimisation cannot be directly achieved, since the quadratic form depends on the unknown functions. Upon multiplying out and omitting the one term which does not involve the parameters, however, the empirical version

3.1 $$\begin{aligned} \begin{aligned} C_n(\theta )&=\displaystyle \int _0^\tau \alpha _{(1)}(s,\theta )^\mathrm{t}V_n(s)\alpha _{(1)}(s,\theta )\,\mathrm{d}s \\&\displaystyle \qquad \quad \,\, -2\int _0^\tau \alpha _{(1)}(s,\theta )^\mathrm{t}V_n(s)\,\mathrm{d}\widetilde{A}_{(1)}(s) \end{aligned} \end{aligned}$$

emerges. Here $\mathrm{d}\widetilde{A}_{(1)}(s)$ contains the first *p* components of the nonparametric $\mathrm{d}\widetilde{A}(s)$ of (2.2), and we let $\widehat{\theta }$ be the minimiser of the criterion function $C_n(\theta )$.

Note that the $V_n(s)$ may very well be data-dependent. We typically have such in mind where $V_n(s)\rightarrow _\mathrm{pr}V(s)$ for a suitable limit matrix function; see the following section, where we also exhibit a particular choice of $V_n(s)$ which leads to optimal performance for large *n*. This involves the nontrivial estimates $1/\{z_i^\mathrm{t}\widetilde{\alpha }(s)\}$, however, discussed in connection with (2.5)–(2.6), and are often too unstable for small and moderate *n*. Our default choice is the simpler

3.2 $$\begin{aligned} V_n(s)=n^{-1}\sum _{i=1}^nY_i(s)z_{i,(1)} z_{i,(1)}^\mathrm{t}, \end{aligned}$$

the upper left $p\times p$ block of $n^{-1}\sum _{i=1}^nY_i(s)z_iz_i^\mathrm{t}$. It has a well-defined limit in probability function *V*(*s*) by Assumptions 1. For the simplest case of having the parametric hazard components constant, with $\alpha _{(1,j)}(s,\theta )=\theta _j$ for $j=1,\ldots ,p$, the above yields

$$\begin{aligned} \widehat{\theta }=\Bigl \{ \int _0^\tau V_n(s)\,\mathrm{d}s\Bigr \}^{-1} \int _0^\tau V_n(s)\,\mathrm{d}\widetilde{A}_{(1)}(s). \end{aligned}$$

These are the best constants, seen as yielding approximations $\widehat{\theta }_j t$ to the nonparametric $\widetilde{A}_{(1,j)}(t)$ for $t\in [0,\tau ]$ and $j=1,\ldots ,p$, as also dictated by the choice of the $V_n(s)$ matrix.

With our default weight function in (3.2), the estimator $\widehat{\theta }$ is similar to the estimator proposed by McKeague & Sasieni (1994, Eq. (2.4), p. 503), but not identical to it. To obtain their estimator, McKeague and Sasieni solve a system of equations obtained by appropriately modifying the score function, obtaining an estimating equation linear in $\theta$ (their $\beta$). Similar techniques may be used with more general parametric hazard functions, thus possibly replacing the $C_n(\theta )$ we work with here with a slightly different criterion function.

### Backfitting to re-estimate the nonparametric part

We now describe a version of Step (b), after Step (a) has yielded parametric estimates $\alpha _j(s,\widehat{\theta })$ for $j=1,\ldots ,p$ as above. Consider the nonparametric part of equation (2.1), that is

$$\begin{aligned} \sum _{i=1}^nw_i(s)z_{i,(2)}\,\mathrm{d}N_i(s)= & {} \sum _{i=1}^nY_i(s)w_i(s)z_{i,(2)}\{z_{i,(1)}^\mathrm{t}\alpha _{(1)}(s,\theta ) +z_{i,(2)}^\mathrm{t}\alpha _{(2)}(s)\}\,\mathrm{d}s \\&+\sum _{i=1}^nw_i(s)z_{i,(2)}\,\mathrm{d}M_i(s). \end{aligned}$$

A more precise definition of the martingales involved, now that work is carried out inside the (1.4) framework, reads

3.3 $$\begin{aligned} M_i(t)=N_i(t)-\int _0^tY_i(s)\{z_{i,(1)}^\mathrm{t}\alpha _{(1)}(s,\theta _0) +z_{i,(2)}^\mathrm{t}\alpha _{(2)}(s)\}\,\mathrm{d}s, \end{aligned}$$

with $\theta _0$ the true parameter. To utilise the parametric knowledge, so as to reach better estimation precision for the nonparametric components, this encourages using

$$\begin{aligned}&\sum _{i=1}^nw_i(s)z_{i,(2)}\{\mathrm{d}N_i(s) -Y_i(s)z_{i,(1)}^\mathrm{t}\alpha _{(1)}(s,\widehat{\theta })\,\mathrm{d}s\}\\&\qquad \quad =\sum _{i=1}^nY_i(s)w_i(s)z_{i,(2)}z_{i,(2)}^\mathrm{t}\,\mathrm{d}\alpha _{(2)}(s) +\mathrm{noise} \end{aligned}$$

to put up

3.4 $$\begin{aligned} \mathrm{d}\widehat{A}_{(2)}(s)=G_{n,22}(s)^{-1} n^{-1}\sum _{i=1}^nw_i(s)z_{i,(2)}\{\mathrm{d}N_i(s) -Y_i(s)z_{i,(1)}^\mathrm{t}\alpha _{(1)}(s,\widehat{\theta })\,\mathrm{d}s\}.\nonumber \\ \end{aligned}$$

This defines modified estimators $\widehat{A}_j(t)$ for $j=p+1,\ldots ,p+q$. Here $G_{n,22}(s)$ is the lower $q\times q$ submatrix of $G_n(s)$.

Note that the method outlined here is really a class of procedures, in that different weight schemes may be used in (3.4), and also different weight functions $V_n$ when minimising the $C_n(\theta )$ function to obtain the $\widehat{\theta }$ estimator. In (3.4), we may e.g. use vanilla weights $w_i(s)=1$, or the more sophisticated $\widetilde{w}_i(s)$ of Sect. 2.2. An asymptotically optimal scheme is found in the next section.

## Large-sample behaviour and optimality

Here we demonstrate limiting normality for the estimators of Sect. 3, i.e. $\widehat{\theta }$ minimising $C_n(\theta )$ of (3.1) and $\widehat{A}_{(2)}(t)$ of (3.4), with precise formulae for the limit distribution variances and covariances. Results are derived under model conditions (1.4), with $\theta _0$ denoting the true parameter for the parametric parts $\alpha _{(1),j}(s,\theta )$ for $j=1,\ldots ,p$. Let $\alpha ^*_{(1)}(s,\theta )$ be the $p\times m$ matrix of first order derivatives $\alpha ^*_j(s,\theta )=\partial \alpha _j(s,\theta )/\partial \theta$ of the *p* component functions, where *m* is the length of the full $\theta$ vector.

### Large-sample theory for the parametric part

For studying our estimators we also need the function *Q*(*s*), defined by

4.1 $$\begin{aligned} Q(s)\,\mathrm{d}s= [G(s)^{-1}\,\mathrm{d}H(s)G(s)^{-1}]_{11}, \end{aligned}$$

that is, the upper left $p\times p$ block matrix of the full $G(s)^{-1}\,\mathrm{d}H(s)G(s)^{-1}$ matrix, associated with the variance of the first *p* components of the Aalen estimator, i.e. $\widetilde{A}_{(1)}$; see (2.4).

#### Proposition 4.1

Suppose regularity conditions spelled out in Assumptions 1 are in force, and that $V_n(s)\rightarrow _\mathrm{pr}V(s)$, uniformly over $s\in [0,\tau ]$. Then $\Lambda _n=\sqrt{n}(\widehat{\theta }-\theta _0)$, under the conditions of the parametric model, tends to $\mathrm{N}_m(0,\Gamma ^{-1}\Omega \Gamma ^{-1})$, in which

$$\begin{aligned} \Gamma= & {} \int _0^\tau \alpha ^*_{(1)}(s,\theta _0)^\mathrm{t}V(s) \alpha ^*_{(1)}(s,\theta _0)\,\mathrm{d}s, \\ \Omega= & {} \int _0^\tau \alpha ^*_{(1)}(s,\theta _0)^\mathrm{t}V(s)Q(s)V(s) \alpha ^*_{(1)}(s,\theta _0)\,\mathrm{d}s. \end{aligned}$$

#### Proof

Setting the derivative of the criterion function (3.1) equal to zero gives the estimation equation $S_n(\widehat{\theta })=0$, where

$$\begin{aligned} S_n(\theta )=\int _0^\tau \alpha ^*_{(1)}(s,\theta )^\mathrm{t}V_n(s) \{\mathrm{d}\widetilde{A}_{(1)}(s)-\alpha _{(1)}(s,\theta )\,\mathrm{d}s\}. \end{aligned}$$

This redefines $\widehat{\theta }$, under appropriate conditions, as an *M*-type estimator; see Hjort (1985, Section 4), Hjort (1992, Section 5). Note that

$$\begin{aligned} \sqrt{n}S_n(\theta _0) \rightarrow _d \int _0^\tau \alpha ^*_{(1)}(s,\theta _0)^\mathrm{t}V(s) [G(s)^{-1}\,\mathrm{d}U(s)]_{(1)}=S, \end{aligned}$$

which at the true $\theta _0$ is a zero-mean normal with variance matrix $\Omega$. A little more work gives expressions for the $m\times m$ matrix $\Gamma _n(\theta )$, containing minus the derivative of $S_n(\theta )$ with respect to the *m* parameters, as

$$\begin{aligned} \Gamma _n(\theta )=\int _0^\tau \alpha ^*_{(1)}(s,\theta )^\mathrm{t}V_n(s) \alpha ^*_{(1)}(s,\theta )\,\mathrm{d}s + E_n(\theta ). \end{aligned}$$

Here the second matrix has components which are linear combinations of smooth and bounded functions of $\theta$ times the *p* components of $\mathrm{d}\widetilde{A}_{(1)}(s)-\alpha _{(1)}(s,\theta )\,\mathrm{d}s$, integrated over $[0,\tau ]$. The point is that all terms of $E_n(\theta )$ go to zero in probability, under model conditions, at $\theta _0$, so $\Gamma _n(\theta _0)\rightarrow _\mathrm{pr}\Gamma$. This leads to $\Lambda _n\rightarrow _d \Gamma ^{-1}S$, proving the claim. $\square$

Asking for the best performance under model conditions, at least for large *n*, is the same as choosing the $p\times p$ matrix function *V* to minimise $\Gamma ^{-1}\Omega \Gamma ^{-1}$. This is achieved when *V*(*s*) is taken proportional to $Q(s)^{-1}$, assuming *Q*(*s*) to have full rank $p\times p$ across the range $[0,\tau ]$. Then $\Gamma =\Omega =\Omega _0$, say, with minimum variance matrix being equal to

4.2 $$\begin{aligned} \Omega _0^{-1}=\Bigl \{\int _0^\tau \alpha ^*_{(1)}(s,\theta _0)^\mathrm{t}Q(s)^{-1}\alpha ^*_{(1)}(s,\theta _0)\,\mathrm{d}s\Bigr \}^{-1}. \end{aligned}$$

To prove that this is the minimum size matrix, let *Z*(*t*) be a Gaußian martingale with incremental variance $\mathrm{Var}\,\mathrm{d}Z(s)=Q(s)\,\mathrm{d}s$, and consider the random vectors $X=\int _0^\tau \alpha ^*_{(1)}V\,\mathrm{d}Z$ and $Y=\int _0^\tau \alpha ^*_{(1)}Q^{-1}\,\mathrm{d}Z$. Their combined variance matrix is

$$\begin{aligned} \Sigma = \begin{pmatrix} \int _0^\tau (\alpha ^*_{(1)})^\mathrm{t}VQV\alpha ^*_{(1)}\,\mathrm{d}s &{}\int _0^\tau (\alpha ^*_{(1)})^\mathrm{t}V\alpha ^*_{(1)}\,\mathrm{d}s \\ \int _0^\tau (\alpha ^*_{(1)})^\mathrm{t}V\alpha ^*_{(1)}\,\mathrm{d}s &{}\int _0^\tau (\alpha ^*_{(1)})^\mathrm{t}Q^{-1}\alpha ^*_{(1)}\,\mathrm{d}s \end{pmatrix}. \end{aligned}$$

In usual block notation, $\Sigma _{11}-\Sigma _{12}\Sigma _{22}^{-1}\Sigma _{21}$ must then be nonnegative definite. This is equivalent to the minimisation claim made.

The next question is how one can make $\Omega _0^{-1}$ as small as possible. But this is the same as minimising over $Q(s)\,\mathrm{d}s=[G(s)^{-1}\,\mathrm{d}H(s)\,G(s)^{-1}]_{11}$, which we have seen takes place for the optimal weights (2.5), and for which we have $Q(s)=[F(s)^{-1}]_{11}=F^{11}(s)$, say. The asymptotically optimal method is accordingly to use as $V_n(s)$ a matrix function which converges in probability, if possible, to $V(s)=F^{11}(s)^{-1}$. But this is achieved via

$$\begin{aligned} V_n(s)=\widetilde{F}_n^{11}(s)^{-1} =\widetilde{F}_{n,11}(s)-\widetilde{F}_{n,12}(s)\widetilde{F}_{n,22}(s)^{-1} \widetilde{F}_{n,21}(s), \end{aligned}$$

where $\widetilde{F}_n$ is as $F_n$ of (2.6), but with weights $z_i^\mathrm{t}\widetilde{\alpha }(s)$ inserted. We may conclude that this method gives the optimal performance for large *n*, with limit variance matrix

4.3 $$\begin{aligned} \Bigl \{\int _0^\tau (\alpha ^*_{(1)})^\mathrm{t}(F^{11})^{-1} \alpha ^*_{(1)}\,\mathrm{d}s\Bigr \}^{-1} =\Bigl \{\int _0^\tau (\alpha ^*_{(1)})^\mathrm{t}(F_{11}-F_{12}F_{22}^{-1}F_{21}) \alpha ^*_{(1)}\,\mathrm{d}s\Bigr \}^{-1}.\nonumber \\ \end{aligned}$$

It is in fact not possible to improve on this, with any other estimation method. That this is indeed so is detailed in Appendix A.

### Large-sample theory for the nonparametric part

To study the behaviour of $\widehat{A}_{p+1},\ldots ,\widehat{A}_{p+q}$ we need the $q\times m$ function

$$\begin{aligned} \phi _n(s)=n^{-1}\sum _{i=1}^nY_i(s)w_i(s)z_{i,(2)}z_{i,(1)}^\mathrm{t}\alpha ^*_{(1)}(s,\theta _0), \end{aligned}$$

which under the mild general conditions stated previously has a limit in probability function $\phi (s)$.

#### Proposition 4.2

Assume that regularity conditions of Proposition 4.1 are in force, and let $\Lambda =\Gamma ^{-1}S$ be the limit variable for $\Lambda _n=\sqrt{n}(\widehat{\theta }-\theta _0)$. Then there is process convergence

4.4 $$\begin{aligned} \sqrt{n}\{\widehat{A}_{(2)}(t)-A_{(2)}(t)\} \rightarrow _d\int _0^t G_{22}(s)^{-1}\,\mathrm{d}U_{(2)}(s) -\int _0^t G_{22}(s)^{-1}\phi (s)\,\mathrm{d}s\,\Lambda \nonumber \\ \end{aligned}$$

in the space $D[0,\tau ]$ of right-continuous functions with left hand limits on $[0,\tau ]$, equipped with the Skorokhod topology.

#### Proof

Some algebra, starting with (3.3) and (3.4), shows that

$$\begin{aligned} \mathrm{d}\widehat{A}_{(2)}(s)= & {} G_{n,22}(s)^{-1} n^{-1}\sum _{i=1}^nw_i(s)z_{i,(2)}[\mathrm{d}M_i(s) +Y_i(s)z_{i,(2)}^\mathrm{t}\alpha _{(2)}(s)\,\mathrm{d}s \\&-\,Y_i(s)z_{i,(1)}^\mathrm{t}\{\alpha _{(1)}(s,\widehat{\theta })-\alpha _{(1)}(s,\theta _0)\}\,\mathrm{d}s], \end{aligned}$$

which leads to

$$\begin{aligned}&\sqrt{n}\{\mathrm{d}\widehat{A}_{(2)}(s)-\alpha _{(2)}(s)\,\mathrm{d}s\}\\&\quad \doteq G_{n,22}(s)^{-1} \Bigl \{n^{-1/2}\sum _{i=1}^nw_i(s)z_{i,(2)}\,\mathrm{d}M_i(s) -\phi _n(s)\,\mathrm{d}s\,\Lambda _n\Bigr \}. \end{aligned}$$

Here $X_n\doteq X_n'$ means that the difference tends to zero in probability. The claim follows from general theory of convergence of processes in the $D[0,\tau ]$ space. $\square$

Propositions 4.1 and 4.2 give clear descriptions of the large-sample behaviour of our parametric and nonparametric estimators, separately. We also need the joint limiting distribution of $\widehat{\theta }$ and $\widehat{A}_{(2)}(t)$, for reaching inference for quantities involving both parts, like the survival curves $S(t\,|\,z)$ with $A(t\,|\,z)=z_1A_1(t,\theta )+\cdots +z_pA_p(t,\theta ) +z_{p+1}A_{p+1}(t)+\cdots +z_{p+q}A_{p+q}(t)$. Here we give details for the joint limiting distribution of $A_{(1)}(t,\widehat{\theta })$ and $\widehat{A}_{(2)}(t)$. We indeed have

4.5 $$\begin{aligned} \sqrt{n}\begin{pmatrix} A_{(1)}(t,\widehat{\theta }) - A_{(1)}(t,\theta _0)\\ \widehat{A}_{(2)}(t) - A_{(2)}(t) \end{pmatrix} \overset{d}{\rightarrow }\mathrm{N}\big (0, \Xi (t)\big ),\quad \text {with}\quad \Xi (t) = \begin{pmatrix} \Xi _{11}(t) &{} \Xi _{12}(t)\\ \Xi _{21}(t) &{} \Xi _{22}(t) \end{pmatrix},\nonumber \\ \end{aligned}$$

with formulae for the variance matrix to follow.

In (4.4), the first term is a Gaußian martingale with variance $\int _0^t G_{22}^{-1}\mathrm{d}H_{22}G_{22}^{-1}$, while the second term also is normal, with a variance which can be written down via Proposition 4.1. By combining Propositions 4.1 and 4.2, and applying the delta method, we reach (4.5). First, $\Xi _{11}(t) = A^{*}(t,\theta _0)\Gamma ^{-1}\Omega \Gamma ^{-1}A^{*}(t,\theta _0)^{\mathrm{t}}$, with $A^{*}(t,\theta )$ being the $p \times m$ matrix with components $\partial A_{j}(t,\theta )/\partial \theta$, for $j = 1,\ldots ,p$. Second, $\Xi _{22}(t)$ is the variance of (4.4). To this end, for the covariance between the two terms in (4.4), we have

4.6 $$\begin{aligned} \begin{array}{rcl} \mathrm{E}\Bigl (\int _0^t G_{22}^{-1}\mathrm{d}U_{(2)}\Bigr )S^\mathrm{t}&{}=&{}\displaystyle \mathrm{E}\Bigl (\int _0^t G_{22}^{-1}\mathrm{d}U_{(2)}\Bigr ) \int _0^\tau (\mathrm{d}U_{(1)}^\mathrm{t}G^{11}+\mathrm{d}U_{(2)}^\mathrm{t}G^{21}) V\alpha ^*_{(1)} \\ &{}=&{}\displaystyle \int _0^tG_{22}^{-1}(\mathrm{d}H_{21}G^{11}+\mathrm{d}H_{22}G^{21}) V\alpha ^*_{(1)}, \end{array} \end{aligned}$$

so that the full variance of the right hand side of (4.4) is

$$\begin{aligned} \Xi _{22}(t)= & {} \int _0^t G_{22}(s)^{-1}\mathrm{d}H_{22}(s)G_{22}(s)^{-1} + \int _0^t G_{22}(s)^{-1}\phi (s)\,\mathrm{d}s\, \Gamma ^{-1}\Omega \Gamma ^{-1}\\&\left( \int _0^t G_{22}(s)^{-1}\phi (s)\,\mathrm{d}s\right) ^{\mathrm{t}} \\&- 2 \int _0^tG_{22}(s)^{-1}\{\mathrm{d}H_{21}(s)G^{11}(s) +\mathrm{d}H_{22}(s)G^{21}(s)\} V(s)\alpha ^*_{(1)}(s)\Gamma ^{-1}\\&\left( \int _0^t G_{22}(s)^{-1}\phi (s)\,\mathrm{d}s\right) ^{\mathrm{t}}. \end{aligned}$$

Third, the lower off-diagonal block in the covariance matrix in (4.5) is

$$\begin{aligned} \Xi _{21}(t)= & {} \mathrm{E}\, \int _0^t G_{22}^{-1}\,\mathrm{d}U_{(2)} S^{\mathrm{t}}\Gamma ^{-1}\,A^{*}(t,\theta _0)^{\mathrm{t}} - \mathrm{E}\, \int _0^t \phi (s)\,\mathrm{d}s\,\Gamma ^{-1}SS^{\mathrm{t}} \Gamma ^{-1}\,A^{*}(t,\theta _0)^{\mathrm{t}} \\= & {} \int _0^t G_{22}^{-1}(\mathrm{d}H_{21}G^{11} + \mathrm{d}H_{22}G^{21})V \alpha _{(1)}^{*}\, \Gamma ^{-1}\,A^*(t,\theta _0)^\mathrm{t}\\&- \int _0^t \phi (s)\,\mathrm{d}s \,\Gamma ^{-1} \Omega \Gamma ^{-1}\,A^{*}(t,\theta _0)^{\mathrm{t}}, \end{aligned}$$

where we use (4.6), and $\Xi _{12}(t) = \Xi _{21}(t)^{\mathrm{t}}$. It is clear how to estimate these covariance matrices, for example, when the traditional Aalen estimator weights $w_i(s)=1$ are being used.

It is interesting to study the special case where $V(s)=F^{11}(s)^{-1}$, which by the above leads to optimal large-sample performance. Then the two terms of the limit process are in fact independent. This follows from $\mathrm{d}H(s)=F(s)\,\mathrm{d}s$ and $G=F$. For this situation, therefore, the covariance function for the limit process in (4.4) may be written

$$\begin{aligned} \int _0^{t_1\wedge t_2}F_{22}(s)^{-1}\,\mathrm{d}s +J(t_1)\Omega _0^{-1}J(t_2)^\mathrm{t}, \end{aligned}$$

where $J(t)=\int _0^tF_{22}^{-1}\phi \,\mathrm{d}s$ and $\phi$ is the limit of

$$\begin{aligned} \phi _n(s)=n^{-1}\sum _{i=1}^nY_i(s)z_{i,(2)} {z_{i,(1)}^\mathrm{t}\alpha ^*_{(1)}(s,\theta _0) \over z_{i,(1)}^\mathrm{t}\alpha _{(1)}(s,\theta _0) +z_{i,(2)}^\mathrm{t}\alpha _{(2)}(s)}. \end{aligned}$$

## Assessing goodness of fit

We have investigated the parametric-nonparametric model (1.4), constructed estimators $\alpha _j(s,\widehat{\theta })$ for the parametric components, and derived large-sample properties, leading to inference methods for all relevant quantities, with better precision than for the traditional nonparametric methods. The underlying assumption for these good results is that the parametric structure actually holds. In this section we construct monitoring processes and related tests to assess adequacy of the parametric part.

### Goodness of fit processes

For each *j* we may consider monitoring processes of the type $\sqrt{n}\int _0^t K_{n,j}(s) \{\mathrm{d}\widetilde{A}_j(s)-\alpha _j(s,\widehat{\theta })\,\mathrm{d}s\}$, where $K_{n,j}$ is a suitable weight function. More generally, let

5.1 $$\begin{aligned} R_n(t)=\sqrt{n}\int _0^t K_n(s) \{\mathrm{d}\widetilde{A}_{(1)}(s)-\alpha _{(1)}(s,\widehat{\theta })\,\mathrm{d}s\}, \end{aligned}$$

with a full $p\times p$ matrix of weight functions $K_{n,ij}(s)$. These processes can be plotted against time to judge the adequacy of the parametric modelling assumptions. The estimator $\widehat{\theta }$ used is as in Sect. 3.1, depending on a matrix weight function $V_n$, for which $\Lambda _n=\sqrt{n}(\widehat{\theta }-\theta )$ under model conditions tends to $\Lambda =\Gamma ^{-1}S\sim \mathrm{N}_m(0,\Gamma ^{-1}\Omega \Gamma ^{-1})$, as defined and derived in Sect. 4.1.

#### Proposition 5.1

Assume that the $K_{n,ij}$ functions are previsible and converge uniformly in probability to $K_{ij}$ functions over $[0,\tau ]$, that regularity conditions associated with the two propositions of Section 4 are in force, and that the parametric model is true for the hazard functions $\alpha _j(s,\theta )$, for $j=1,\ldots ,p$. Then there is process convergence in the space $D([0,\tau ]^p)$, equipped with the Skorokhod product topology, and

$$\begin{aligned} R_n(t)\rightarrow _d R(t)=\int _0^t K[G^{-1}\,\mathrm{d}U]_{(1)} -\int _0^t K\alpha ^*_{(1)}\,\mathrm{d}s\,\Lambda . \end{aligned}$$

#### Proof

Letting as before $\alpha _j^*(s,\theta )=\partial \alpha _j(s,\theta )/\partial \theta$, and using the representation (2.4) with $\mathrm{d}U_n(s)$ of (2.3), the essence here is that

$$\begin{aligned} \sqrt{n}\{\mathrm{d}\widetilde{A}_j(s)-\alpha _j(s,\widehat{\theta })\} \doteq [G_n(s)^{-1}\,\mathrm{d}U_n(s)]_j -\alpha ^*_j(s,\theta )^\mathrm{t}\sqrt{n}(\widehat{\theta }-\theta )\,\mathrm{d}s \end{aligned}$$

for $j=1,\ldots ,p$, by Taylor analysis. It follows from methods and results of Sect. 4 that there is joint distributional convergence of $G_n(s)^{-1}\,\mathrm{d}U_n(s)$ and $\sqrt{n}(\widehat{\theta }-\theta )$ to $G(s)^{-1}\,\mathrm{d}U(s)$ and $\Lambda =\Gamma ^{-1}S$. Let us write $W(t)=\int _0^t [G(s)^{-1}\,\mathrm{d}U(s)]_{(1)}$, so that $\mathrm{d}W(s)=G^{11}(s)\,\mathrm{d}U_{(1)}(s)+G^{12}(s)\,\mathrm{d}U_{(2)}(s)$. We then have

$$\begin{aligned} \sqrt{n}\{\mathrm{d}\widetilde{A}_{(1)}(s)-\alpha _{(1)}(s,\widehat{\theta })\} \rightarrow _d \mathrm{d}W(s)-\alpha _{(1)}^*(s,\theta ) \Gamma ^{-1} \int _0^\tau \alpha _{(1)}^*(s,\theta _0)^\mathrm{t}V(s)\,\mathrm{d}W(s). \end{aligned}$$

With the weight functions $K_n(s)$ converging uniformly in probability to the *K*(*s*), we reach $R_n\rightarrow _d R$ via details and methods similar to those used in Hjort (1990, Sections 3–4), for a similar though somewhat different setup. $\square$

The limiting processes $R_1,\ldots ,R_p$ are jointly Gaußian with zero mean. To find their covariance functions we utilise the structure found in the proof of the proposition. The *W* process is a normal martingale with independent increments, and $\mathrm{Var}\,\mathrm{d}W(s)=Q(s)\,\mathrm{d}s$, as before, see (4.1). Then

$$\begin{aligned} R(t)=\int _0^t K\,\mathrm{d}W-\Psi (t)\Lambda , \quad \mathrm{with} \quad \Lambda =\Gamma ^{-1}\int _0^\tau (\alpha ^*_{(1)})^\mathrm{t}V\,\mathrm{d}W, \end{aligned}$$

writing also $\Psi (t)$ for the $p\times m$ matrix $\int _0^tK\alpha ^*_{(1)}\,\mathrm{d}s$. Taking the mean of

$$\begin{aligned} R(t_1)R(t_2)^\mathrm{t}= & {} \int _0^{t_1}K\,\mathrm{d}W\int _0^{t_1}\mathrm{d}W^\mathrm{t}K^\mathrm{t}+\Psi (t_1)\Lambda \Lambda ^\mathrm{t}\Psi (t_2)^\mathrm{t}\\&-\int _0^{t_1}K\,\mathrm{d}W\,\Lambda ^\mathrm{t}\Psi (t_2)^\mathrm{t}-\Psi (t_1)\Lambda \int _0^{t_2}\mathrm{d}W^\mathrm{t}K^\mathrm{t}, \end{aligned}$$

using the zero-mean independent increments property of *W*, gives

$$\begin{aligned} \int _0^ {t_1\wedge t_2}KQK^\mathrm{t}\,\mathrm{d}s +\Psi (t_1)\Gamma ^{-1}\Omega \Gamma ^{-1}\Psi (t_2)^\mathrm{t}-\Phi (t_1)\Gamma ^{-1}\Psi (t_2)^\mathrm{t}-\Psi (t_1)\Gamma ^{-1}\Phi (t_2)^\mathrm{t}, \end{aligned}$$

where $\Phi (t)$ is the $p\times m$ matrix function $\int _0^t KQV\alpha ^*_{(1)}\,\mathrm{d}s$.

The (5.1) framework involves a full matrix of weight functions and gives *p* processes for simultaneous monitoring. We note the special case of a single $p\times 1$ weight function $K_n=(K_{n,1},\ldots ,K_{n,p})^\mathrm{t}$, where a result can be read off from those above, by considering only one monitoring process. So, the linear combination of compared increments

$$\begin{aligned} R_n^*(t)= & {} \sqrt{n}\int _0^t K_n(s)^\mathrm{t}\{\mathrm{d}\widetilde{A}_{(1)}(s) -\alpha _{(1)}(s,\widehat{\theta })\,\mathrm{d}s\} \\= & {} \sqrt{n}\sum _{j=1}^k \int _0^t K_{n,j}(s)\{\mathrm{d}\widetilde{A}_j(s) -\alpha _j(s,\widehat{\theta })\,\mathrm{d}s\} \end{aligned}$$

converges in distribution as a process to $R^*(t)=\int _0^t K^\mathrm{t}\,\mathrm{d}W-\psi (t)\Lambda$, where now $\psi (t)=\int _0^tK^\mathrm{t}\alpha ^*_{(1)}\,\mathrm{d}s$.

If in particular $K_n=(0,\ldots ,K_{n,j},\ldots ,0)^\mathrm{t}$, we are led to the separate monitoring processes

5.2 $$\begin{aligned} R_{n,j}(t)=\sqrt{n}\int _0^t K_{n,j}(s) \{\mathrm{d}\widetilde{A}_j(s)-\alpha _j(s,\widehat{\theta })\,\mathrm{d}s\}, \quad \mathrm{for\ }j=1,\ldots ,p. \end{aligned}$$

This $R_{n,j}(t)$ tends in distribution to

5.3 $$\begin{aligned} R_j(t)=\int _0^t K_j(s)\,\mathrm{d}W_j(s)-\psi _j(t)^\mathrm{t}\Gamma ^{-1}S, \quad \mathrm{with} \quad S=\int _0^\tau (\alpha _{(1)}^*)^\mathrm{t}V\,\mathrm{d}W, \end{aligned}$$

where $\psi _j(t)=\int _0^tK_j (\alpha ^*_j)^\mathrm{t}\,\mathrm{d}s$ (of size $m\times 1$). With calculations similar to those above, the covariance function $\mathrm{cov}\{R_j(t_1),R_j(t_2)\}$ may be expressed as

5.4 $$\begin{aligned} \begin{aligned}&\displaystyle \int _0^{t_1\wedge t_2} K_j^2Q_{jj}\,\mathrm{d}s +\psi _j(t_1)^\mathrm{t}\Gamma ^{-1}\Omega \Gamma ^{-1}\psi _j(t_2) \\&\qquad \qquad \displaystyle - \, \psi _j(t_1)^\mathrm{t}\Gamma ^{-1}\Phi _j(t_2) -\psi _j(t_2)^\mathrm{t}\Gamma ^{-1}\Phi _j(t_1), \end{aligned} \end{aligned}$$

where

$$\begin{aligned} \Phi _j(t) =\mathrm{E}\int _0^\tau (\alpha _{(1)}^*)^\mathrm{t}V\,\mathrm{d}W\int _0^t K_j\,\mathrm{d}W_j =\int _0^t K_j(s)\alpha _{(1)}^*(s,\theta _0)^\mathrm{t}V(s) Q^{(j)}(s)\,\mathrm{d}s, \end{aligned}$$

writing $Q^{(j)}(s)$ for column *j* of the $p\times p$ matrix *Q*(*s*). Like $\psi _j(t)$, the $\Phi _j(t)$ is of size $m\times 1$.

### Chi-squared tests

Divide the time observation period $[0,\tau ]$ into time windows $I_\ell =(c_{\ell -1},c_\ell ]$ for $\ell =1,\ldots ,k$, where $c_0=0$ and $c_k=\tau$. For each window we may compute the *p*-variate increment $\Delta R_n(I_\ell )=R_n(c_\ell )-R_n(c_{\ell -1})$. From Proposition 5.1, the collection of these tends in distribution to that of $\Delta R(I_\ell )=R(c_\ell )-R(c_{\ell -1})$, which under the model hypothesis is zero-mean multinormal and with a covariance structure which might be calculated from the above results.

We may somewhat grandly test the full simultaneous parametric hypothesis that all $\alpha _j(s,\theta )$ components hold, via the *p*-dimensional $\Delta R_n(I_j)$. Here we outline simpler but natural strategies connected to studying one $\alpha _j(s,\theta )$ at the time. For this we use $R_{n,j}(t)\rightarrow _d R_j(t)$, as per (5.2)–(5.3), for a given choice of weight function $K_{n,j}(s)$. We compute increments $\Delta R_{n,j,\ell }=R_{n,j}(I_\ell )$, and these tend jointly to the vector of increments $\Delta R_j(I_\ell )= \Delta \{R_j(c_\ell )-R_j(c_{\ell -1})\}$. This is a zero-mean multinormal, say $\mathrm{N}_k(0,\Sigma _j)$, with $\Sigma _j$ the appropriate covariance matrix flowing from the covariance function (5.4). There are several ways in which we may now test the $\alpha _j(s,\theta )$ hypothesis. In particular,

5.5 $$\begin{aligned} C_{n,j}=\Delta _{n,j}^\mathrm{t}\widehat{\Sigma }_j^{-1}\Delta _{n,j} \rightarrow _d C_j=\Delta _j^\mathrm{t}\Sigma _j^{-1}\Delta _j\sim \chi ^2_k, \end{aligned}$$

where $\Delta _{n,j}$ is the vector of the $\Delta R_{n,j}$, tending in distribution to $\Delta _j$, the vector of the $\Delta R_j(I_\ell )$, and $\widehat{\Sigma }_j$ a consistent estimator of the $k\times k$ matrix $\Sigma _j$.

### Other tests

It is in principle easy to construct other test statistics based on the monitoring processes $R_n$ of (5.1), although their exact or limiting null distributions might be hard to tabulate or assess. There are ways of approximating such distributions, however, as we now illustrate. Consider $R_{n,j}$ of (5.2), for a suitable $K_{n,j}$, and define

$$\begin{aligned} \Vert R_{n,j}\Vert =\max _{t\le \tau }|R_{n,j}(t)| \quad \mathrm{for\ }j=1,\ldots ,p. \end{aligned}$$

These Kolmogorov–Smirnov type tests have well-defined limit distributions, namely $\max _{t\le \tau } |R_j(t)|$ with $R_j(t)$ as in (5.3), a process defined in terms of $W(t)=\int _0^t [G(s)^{-1}\,\mathrm{d}U(s)]_{(1)}$. Options for deciding on an upper critical point in the null distribution of $\Vert R_{n,j}\Vert$ include the following: (i) One may simulate from the limit distribution, at the estimated versions of $K_j$, *Q* and $\alpha ^*_j(s,\theta )$. This can be done with relative ease by simulating *W* processes, via independent normal increments. (ii) One may simulate from the $\Vert R_{n,j}\Vert$ distribution, again at its estimated position with respect to $K_j$, *Q*, and $\alpha ^*_j$, by simulating full $N_i^*$ and $Y_i^*$ processes from the model where the *i*th life-time comes from the distribution with integrated hazard rate $z_{i,(1)}^\mathrm{t}A_{(1)}(t,\widehat{\theta })+z_{i,(2)}^\mathrm{t}\widehat{A}_{(2)}(t)$. This amounts to semiparametric bootstrapping at the estimated model.

Note that the above methods also apply to the simultaneous test statistic $\sum _{j=1}^k\Vert R_{n,j}\Vert$, and relatives thereof.

## Simulations and an application

In this section we compare the fully nonparametric linear hazard regression model, that is, the Aalen model, with the partly parametric partly nonparametric linear hazard regression model developed in this paper. First, in Sect. 6.1, this comparison takes place on simulated data; while Sect. 6.2 contains an analysis of $n = 312$ Primary biliary cirrhosis patients that participated in a double-blind randomised study at the Mayo Clinic in the USA between January 1974 and May 1984. This dataset is contained in the R package survival (Therneau and Lumley 2013).

**Fig. 1:**
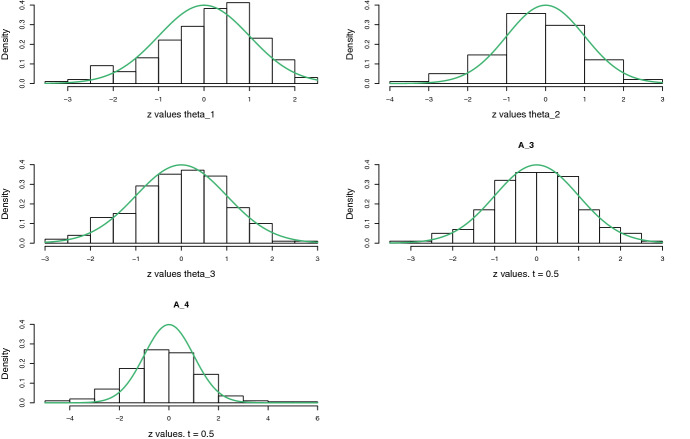
Histograms of $\sqrt{n}(\widehat{\theta }_k - \theta _k)/\mathrm{se}(\widehat{\theta }_k)$ and $\sqrt{n}\{\widehat{A}_j(t) - A_j(t)\}/\mathrm{se}(\widehat{A}_j(t))$ for $k = 1,2,3$, and $j = 3,4$. The cumulative regressors are evaluated at $t = 0.5$. The sample size was set to $n = 2000$, and the histograms are based on 200 simulations. The green curves indicate the standard normal density (Color figure online)

### Simulations

We simulated 200 datasets of $n = 2000$ potentially right-censored survival times, with the covariates held fixed across the 200 simulations (reflecting that the large-sample theory of this paper is developed conditionally on the covariates, see Assumptions 1). The true hazard rate of the *i*th individual was taken to be $h_i(t) = \theta _1\theta _2 t^{\theta _2 - 1}z_{i,1} + \theta _3 t z_{i,2} + 0.572\,t^{2 - 1} + 0.123\, t z_{i,4}$, with $\theta _1 = 0.123$, $\theta _2 = 2$, and $\theta _3 = 0.567$. The censoring times were drawn from the uniform distribution on [0, 1], resulting in about 55 percent of the survival times being observed. To each data set we fit the Aalen linear hazard regression model with four regressors, and also a correctly specified partly parametric partly nonparametric model, that is, the model with hazard rate

$$\begin{aligned} h_i(t) = \theta _1\theta _2 t^{\theta _2 - 1}z_{i,1} + \theta _3 t z_{i,2} + \alpha _{3}(t) + \alpha _{4}(t)z_{i,4}, \end{aligned}$$

meaning that $\alpha _{3}(t)$ is the ‘intercept’ function. Figure 1 displays histograms of the *z*-values (or Wald statistics) $\sqrt{n}(\widehat{\theta }_k - \theta _k)/\mathrm{se}(\widehat{\theta }_k)$ for $k = 1,2,3$, and $\sqrt{n}\{\widehat{A}_j(t) - A(t)\}/\mathrm{se}(\widehat{A}_j(t))$ for $j = 3,4$, the latter evaluated at time $t = 0.5$. The standard errors $\mathrm{se}(\widehat{\theta }_k)$ and $\mathrm{se}(\widehat{A}_j(t))$ used to compute these statistics are estimates of the true standard deviations of the estimators. The histograms indicate the with $p = q = 2$ and $m =3$, a rather large sample size is needed for the normality to really kick in for all estimands.

**Fig. 2:**
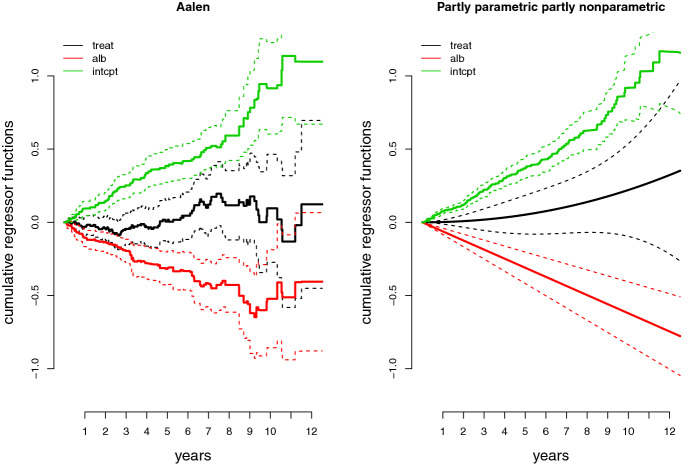
Estimates of the cumulative regression functions in (6.1), fitted to the PBC-data set. The dashed lines indicate pointwise approximate 95 percent confidence bands

### Empirical application

Primary biliary cirrhosis (PBC) is a rare but serious liver disease of unknown origin. Between January 1974 and May 1984, 312 PBC-patients were included in a double-blind randomized study at the Mayo Clinic in the USA, comparing D-penicillamine with placebo. In our analysis, we have chosen to model the hazard rate of the *i*th patient as

6.1 $$\begin{aligned} h_i(t) = \alpha _1(t)\,\texttt {treat}_i + \alpha _2(t)\, \texttt {alb}_i + \alpha _{3}(t), \end{aligned}$$

where $\texttt {treat}_i$ is an indicator taking the value zero if placebo, and one if D-penicillamine; and $\texttt {alb}_i$ is the concentration of serum albumin (in $\text {g}/\text {dl}$) of the *i*th patient. The covariate $\texttt {alb}_i$ was centred around its mean, and standardised by its standard deviation. We estimated the cumulatives $A_j(t) = \int _0^t \alpha _j(s)\,\mathrm{d}s$, both by using the Aalen estimator $\widetilde{A}(t)$ of (2.2); and by parametrising the regression functions $\alpha _1(t)$ and $\alpha _2(t)$ as $\alpha _1(t) = \alpha _1(t,\theta ) = \theta _1 \theta _2 t^{\theta _2 - 1}$, and $\alpha _2(t) = \alpha _2(t,\theta ) = \theta _3 t$, and using the estimation methods developed in this paper.

**Fig. 3:**
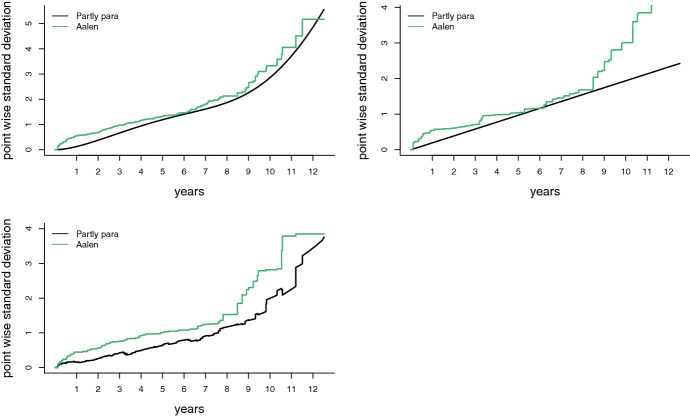
Estimated pointwise standard deviations of the estimators $\widetilde{A}_j(t)$ for $j =1,2,3$ of the Aalen model (in green), and $A_1(t,\widehat{\theta })$, $A_2(t,\widehat{\theta })$, and $\widehat{A}_3(t)$, of the partly parametric partly non-parametric model (in black) (Color figure online)

The estimated cumulative regression functions, along with pointwise approximate 95 percent confidence bands, are plotted in Fig. 2. For the parametric cumulative regressors the confidence bands were obtained by an application of the delta method, and using Proposition 4.1. From the two plots in Fig. 2, it is not easy to see that the confidence bands for the estimators in the partly parametric partly nonparametric model are more narrow than those of the Aalen model. In Fig. 3, therefore, we have plotted the estimated pointwise standard deviations for all six estimators of the cumulative regression functions, clearly showing the gains in efficiency.

In order to make a stab at assessing the goodness of fit of the parametric functions, Fig. 5 displays the $R_{n,1}(t)$ and $R_{n,2}(t)$ functions of (5.2), as developed in Sect. 5. In particular, the blue line shows $\sqrt{n}(\widehat{A}_1(t) - \widehat{\theta }_1 t^{\widehat{\theta }_2})$, while the green line shows $\sqrt{n}(\widetilde{A}_2(t) - \widehat{\theta }_3 t)$. We see that the parametric regressors seem to give a decent fit for the first eight years in the data, while for the remaining years the Aalen estimators and the parametric estimates diverge somewhat. One should keep in mind, however, that with $n = 312$, the amount of data we have for these later years is rather limited, which means increasing variance for $\widetilde{A}_1(t)$ and $\widetilde{A}_2(t)$. A formal test for the adequacy of the parametric hazard functions may be carried out using the apparatus of Sect. 5.2.

**Fig. 4:**
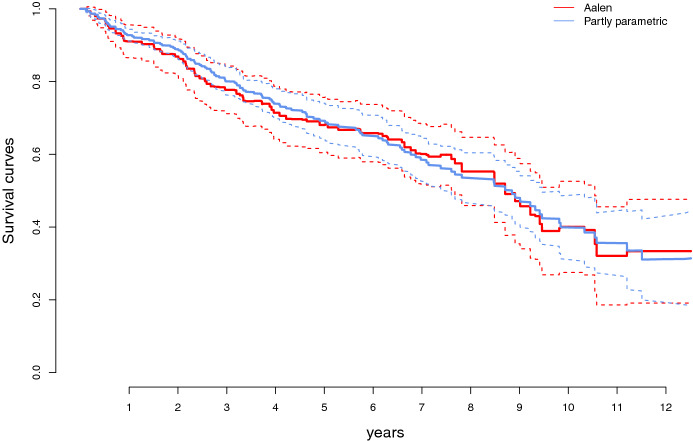
The estimated survival curves corresponding to the estimated cumulative regression functions plotted in Fig. 2, for a non-treated individual with $\texttt {alb}_i$ equal to its mean. The dashed lines indicate approximate 95 percent confidence bands

Figure 4 displays the estimated survival curves of an individual, corresponding to the Aalen, and the partly parametric partly nonparametric linear hazard regression model, respectively, along with pointwise approximate 95 percent confidence bands (see Sect. B.3). The two survival curves in Fig. 4 follow each other closely, but the confidence band for the partly parametric partly nonparametric model is always tighter than that corresponding to the Aalen estimator.

**Fig. 5:**
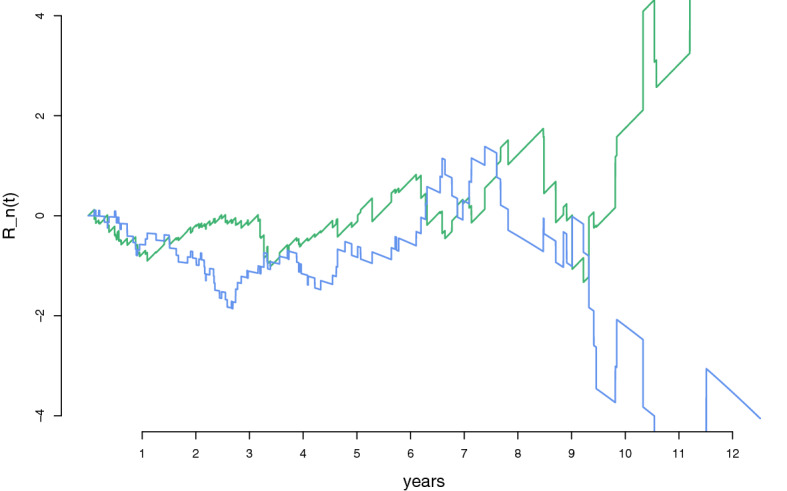
The $R_{n,j}(t)$ functions of (5.1), with weight functions $K_n(t) = 1$. The blue line shows $\sqrt{n}(\widetilde{A}_1(t) - \widehat{\theta }_1 t^{\widehat{\theta }_2})$, while the green line shows $\sqrt{n}(\widetilde{A}_2(t) - \widehat{\theta }_3 t)$ (Color figure online)

## Concluding remarks

We end our article with a list of concluding remarks, some pointing to further research.

**A. Link to GAM.** We have investigated parametric-nonparametric models for the Aalen hazard function model $\sum _{j=1}^r z_j\alpha _j(s)$. There are similarities to the generalised additive regression models, where the mean response curve for covariates $x_1,\ldots ,x_r$ is modelled as $\mathrm{E}\,(Y\,|\,x_1,\ldots ,x_r)=f_1(x_1)+\cdots +f_r(x_r)$. The typical GAM machinery takes the regression functions $f_j(x_j)$ functions to be nonparametric, where there are several estimation methods; see Hastie and Tibshirani (1990); Wood (2017). Methods of the present paper may inspire parametric-nonparametric versions of GAM, with some of the $f_j(x_j)$ modelled parametrically.

**B. Local power of goodness-of-fit tests.** The monitoring functions of Sect. 5, i.e. the $R_n(t)$ and $R_{n,j}(t)$, lead as explained there to classes of goodness-of-fit tests, including chi-squared and Kolmogorov–Smirnov type versions. One may also investigate the local power of such tests, by extending Proposition 5.1 to the situation where the true $\alpha _j(s)$ functions are $O(1/\sqrt{n})$ away from parametric $\alpha _j(s,\theta _0)$. Such results may then be used further for constructing weight functions $K_n(s)$ with optimal local power against certain envisaged alternatives.

**C. Large-sample behaviour outside model conditions.** In Sect. 4 clear limiting normality results have been derived under model assumptions. These may be extended to situations where the real underlying hazard function structure takes the general Aalen form $\sum _{j=1}^r z_j\alpha _j(s)$, with the first *p* of the $\alpha _j(s)$ not necessarily being inside the parametric models, say $\alpha _j(s,\theta _j)$. This involves certain least false parameters $\theta _{0,j}$. The benefit of having such more general outside-model results is partly to construct model robust methods for confidence intervals, etc., and also for building appropriate model selection strategies.

**D. FIC for model selection.** Our parametric-nonparametric model machinery has been developed for a given set of parametric model components, say $\alpha _j(s,\theta _j)$ for components $j=1,\ldots ,p$. It would clearly be useful to develop supplementing model selection methodology, for situations where the statistician is not able or willing to decide a priori which components to take parametric, and in that case which parametric structures to use. Methods of the AIC and BIC variety cannot be used, since there are no likelihood functions. One may however develop FIC methods, for the Focused Information Criterion; see Claeskens and Hjort (2008, Ch. 6–7) for a general discussion. FIC methods along the lines developed in Jullum and Hjort (2019), Claeskens et al. (2019) can be constructed in the present setup. The start assumption is that the nonparametric Aalen model holds, for certain unknown $\alpha _j(t)$ for $j=1,\ldots ,r$. For a given quantity of interest, say $\mu =\mu (\alpha _1(\cdot ),\ldots ,\alpha _r(\cdot ))$, there would be a list of ensuing estimators, say $\widehat{\mu }_M$ for candidate model *M*. The FIC would then be an estimator of the mean squared error for these $\widehat{\mu }_M$. Carrying out this would need large-sample normality results outside parametric model conditions, as briefly pointed to in point D above.

**E. Alternative estimation strategies.** Our estimators for the parametric-nonparametric model use for Step (a) minimisation of a certain criterion function $C_n(\theta )$ of (3.1), with the resulting $\widehat{\theta }$ also being used in Step (b) for the nonparametric components. Other strategies may also be used for Step (a), including minimising other criterion functions for making $\alpha _{(1)}(s,\theta )$ come close to the underlying $\alpha _{(1)}(s)$. Special versions of such ideas lead to M-type estimators, for which theory is given in Hjort (1985, Section 4). Extending the full theory to estimation of both $\theta$ and $A_{(2)}(t)$ takes further efforts, however.

**F. A parametric-nonparametric cure model.** In recent years, cure models have gained much attention. See Amico and Van Keilegom (2018) for a review, and the references therein. These are models for survival times where an unknown fraction of the population under study is ‘cured’, in the sense that the individuals belonging to this fraction will never experience the event of interest. The population survival curve for the (standard) cure model takes the form $S_\mathrm{pop.}(t) = 1 - \pi + \pi S(t)$, where *S*(*t*) is a proper survival function (that is, $S(t) \rightarrow 0$ as $t \rightarrow \infty$), and $\pi$ is the probability of being susceptible to the event of interest. Both *S*(*t*) and $\pi$ are typically modelled as functions of covariates, $S(t) = S(t \,|\,z)$ and $\pi = \pi (x^{\mathrm{t}}\gamma )$, where *z* and *x* are potentially different sets of covariates. In Stoltenberg (2020) a cure model with a linear hazard regression model à la Aalen is introduced, as in 1.3 the (proper) survival function takes the form $S(t\,|\,z) = \exp \{-z^{\mathrm{t}}A(t)\}$, and estimation methods for the $A_j(t)$ as well as the parameters entering $\pi (x^{\mathrm{t}}\gamma )$ are developed. Inspired by the development of the present paper, estimation methods and accompanying large-sample theory could be developed for the partly parametric partly nonparametric cure model, that is, a model whose population survival function is

$$\begin{aligned} S_\mathrm{pop.}(t;x,z)= & {} 1 - \pi (x^{\mathrm{t}}\gamma )\\&+ \pi (x^{\mathrm{t}}\gamma ) \exp \Bigl [-\int _0^t\Big \{\sum _{j=1}^p z_{i,j}\alpha _j(s,\theta ) + \sum _{j=p+1}^{p+q}z_{i,j}\alpha _j(s) \Big \}\,\mathrm{d}s\Bigr ], \end{aligned}$$

with $\pi (a) :\mathbb {R}\rightarrow [0,1]$ some parametric function, for example the logistic one. The unknowns of this model, that need to be estimated from the data, are the parameter vectors $\gamma$ and $\theta$, as well as the nonparametric cumulatives $A_{j}(t) = \int _0^t \alpha _j(s)\,\mathrm{d}s$ for $j = p+1,\ldots ,p+q$. Estimators for these may be obtained by combining the estimators developed in Stoltenberg (2020) with the two-step estimation procedure of the present paper.

## Asymptotic optimality

Consider the family of models whose hazard rates are

A.1 $$\begin{aligned} \begin{aligned} h_i(t,\theta ,\eta )&= z_{i,(1)}^{\mathrm{t}}\alpha _{(1)}(t,\theta ) + z_{i,(2)}^{\mathrm{t}}\alpha _{(2)}(t,\eta ) \\&= \sum _{j=1}^p z_{i,j}\alpha _{j}(t,\theta ) + \sum _{j=1}^{p+q} z_{i,j} \alpha _j(t,\eta ), \end{aligned} \end{aligned}$$

where $$\alpha _j(t,\eta ) = \sum _{l=1}^K \eta _{j,l} I_{W_l}$$, with $$W_{l} = [v_{l-1},v_l)$$, for an equidistant partition $$0 = v_0< v_1< \cdots< v_{K-1} < v_{K} = \tau $$ of the observational window $$[0,\tau ]$$. We assume that $$\alpha _j(s,\theta _j)$$ for $$j = 1,\ldots ,p$$, so that $$r \ge p$$. If $$\theta \in \mathbb {R}^r$$ say, then (A.1) is a $$r + qK$$ dimensional model. This is now a fully parametric model, so we can use theory from Sect. 2.3. Until we say otherwise, we are going to assume that the true model is of the form (A.1), *with K held fixed*. The log-likelihood function is $$\ell _n(\theta ,\eta ) = \sum _{i=1}^n\int _0^{\tau }\{\log h_i(s,\theta ,\eta ) \,\mathrm{d}N_i(s) - Y_i(s)h_i(s,\theta ,\eta )\,\mathrm{d}s\}$$. We split the score function in a $$\theta $$-part and an $$\eta $$-part. We have

$$\begin{aligned} U_{n} = n^{-1/2}\sum _{i=1}^n \int _0^\tau \frac{\alpha _{(1)}^{*}(s,\theta )^{\mathrm{t}}z_{i,(1)}}{h_i(s,\theta ,\eta )}\,\mathrm{d}M_i(s) \end{aligned}$$

which is an $r \times 1$ column vector (with *r* being the dimension of $\theta$), and where $\alpha _{(1)}^{*}$ is a $p \times r$ matrix containing the partial derivatives $\partial \alpha _{j}(s,\theta _j)/\partial \theta _j$ for $j = 1,\ldots ,p$. We also have the score function $V_n = (V_{n,1}^{\mathrm{t}},\ldots ,V_{n,K}^{\mathrm{t}})^{\mathrm{t}}$, which is a $qK \times 1$ column vector, where

$$\begin{aligned} V_{n,l} = n^{-1/2}\sum _{i=1}^n \int _{W_l} \frac{z_{i,(2)}}{h_i(s,\theta ,\eta )}\,\mathrm{d}M_i(s)\qquad \text {for } l = 1,\ldots ,K, \end{aligned}$$

are $q \times 1$ column vectors. Let $J_n$ be the variance process of $(U_n,V_n)$, and let $(\widehat{\theta },\widehat{\eta })$ be the maximum likelihood estimator. Under the conditions of Proposition 2.1, we know that $\sqrt{n}(\widehat{\theta } - \theta _0,\widehat{\eta } - \eta _{0,K})$ converges in distribution to $\mathrm{N}_{r + qK}(0,\Omega _{K}^{-1})$ as $n \rightarrow \infty$, where $\Omega _{K}$ is the limit in probability of $J_n$, and $\theta _0,\eta _{0,K}$ denote the true values of the parameters (under the *K*’th model). We need to find the limiting distribution of $\widehat{\theta }$ and the estimator for the cumulative $A_2(t,\eta ) = \int _0^t \alpha _2(s,\eta )\,\mathrm{d}s$. Introduce the $(p+q) \times (p+qK)$ matrix function $H_t:\mathbb {R}^{p+qK} \rightarrow \mathbb {R}^{p+q}$ that is such that $H_t (\theta ^{\mathrm{t}},\eta ^{\mathrm{t}})^{\mathrm{t}} = (\theta _1,\ldots ,\theta _r,A_{p+1}(t,\eta ),\ldots ,A_{p+q}(t,\eta ))^{\mathrm{t}}$ for each *t*. (The function $H_t$ also depends on *K*, but we suppress this from the notation as it is of little relevance in the following.) An application of the delta-method now yields

$$\begin{aligned} \sqrt{n}\{\widehat{\theta } - \theta , A_2(\tau ,\widehat{\eta }) - A_2(\tau ,\eta _{0,K}) \} \overset{d}{\rightarrow }\mathrm{N}_{p+q}\{0, H_{\tau }\Omega _{K}^{-1} H_{\tau }^{\mathrm{t}} \}. \end{aligned}$$

(The full process convergence version of this result is not necessary for what we are about to show.) The upper left block of $H_{\tau }\Omega _{K}^{-1}H_{\tau }^{\mathrm{t}}$ is the $r \times r$ (limiting) variance matrix of $\sqrt{n}(\widehat{\theta } - \theta _0)$. Using the notation

$$\begin{aligned} \Omega _{K} = \begin{pmatrix} \Omega _{K,11} &{} \Omega _{K,12}\\ \Omega _{K,21} &{} \Omega _{K,22} \end{pmatrix} \quad \text {and}\quad \Omega _K^{-1} = \begin{pmatrix} \Omega _K^{11} &{} \Omega _K^{12}\\ \Omega _K^{21} &{} \Omega _K^{22} \end{pmatrix}, \end{aligned}$$

the upper left block of $H_{\tau }\Omega _{K}^{-1}H_{\tau }^{\mathrm{t}}$ is

$$\begin{aligned} \Omega _K^{11} = (\Omega _{K,11} - \Omega _{K,12}\Omega _{K,22}^{-1}\Omega _{K,21})^{-1}. \end{aligned}$$

By doing the matrix algebra, we see that the matrices inside the parentheses are the $r \times r$ matrix

$$\begin{aligned} \Omega _{K,11} = \int _0^{\tau }(\alpha _{(1)}^*)^{\mathrm{t}} F_{11}\alpha _{(1)}^*\,\mathrm{d}s, \end{aligned}$$

the $$r \times qK$$ matrix

$$\begin{aligned} \Omega _{K,12} = \int _0^{\tau }[(\alpha _{(1)}^*)^{\mathrm{t}}F_{12}I_{W_1} \cdots (\alpha _{(1)}^*)^{\mathrm{t}}F_{12}I_{W_K}]\,\mathrm{d}s, \end{aligned}$$

and $\Omega _{K,21} = \Omega _{K,12}^{\mathrm{t}}$, while $\Omega _{K,22}$ is the $qK\times qK$ block diagonal matrix whose blocks are the $q\times q$ matrices

$$\begin{aligned} (\Omega _{K,22})_{l} = F_{22}I_{W_l},\quad \text {for } l = 1,\ldots ,K. \end{aligned}$$

Here, $F_{11},F_{12} = F_{21}$ and $F_{22}$ are the probability limits of $F_{n,11},F_{n,12} = F_{n,21}$ and $F_{n,22}$, respectively, where these latter are the blocks of the matrix $F_n$ defined in (2.6). We now consider $\Omega _K$ as $K\rightarrow \infty$, that is, as the interval lengths shrink to zero. Under appropriate conditions on the covariates, on the probability limit of $n^{-1}\sum _{i=1}^n Y_i(s)$, and on the function $s \mapsto \alpha _{(1)}^*$ (e.g. bounded derivatives), we have that the *l*’th diagonal block of $\Omega _{K,22}$ is

$$\begin{aligned} (\Omega _{K,22})_{l} = F_{22}(v_{l-1})K^{-1} + O(K^{-2}), \quad \text {for } l = 1,\ldots ,K, \end{aligned}$$

and, similarly,

$$\begin{aligned} \Omega _{K,12} = [(\alpha _{(1)}^*)^{\mathrm{t}}F_{12}(v_0) \cdots (\alpha _{(1)}^*)^{\mathrm{t}}F_{12}(v_{K-1})]K^{-1} + O(K^{-2}). \end{aligned}$$

We then get

$$\begin{aligned}&\Omega _{K,12}\Omega _{K,22}^{-1}\Omega _{K,21}\\&\quad = K^{-1}\sum _{l=1}^K (\alpha _{(1)}^{*}(v_{l-1}))^{\mathrm{t}}F_{12}(v_{l-1})F_{22}(v_{l-1})^{-1}F_{21}(v_{l-1})\alpha _{(1)}^{*}(v_{l-1}) + O(K^{-2}), \end{aligned}$$

which is a Riemann sum converging to $\int _0^{\tau } (\alpha _{(1)}^{*})^{\mathrm{t}}F_{12}F_{22}^{-1}F_{21}\alpha _{(1)}^{*}\,\mathrm{d}s$ as $K \rightarrow \infty$. In conclusion, $J_n \rightarrow _p \Omega _K$ as $n \rightarrow \infty$ with *K* fixed, and

$$\begin{aligned} \Omega _{K,11}^{-1} \rightarrow \Big \{\int _0^{\tau } (\alpha _{(1)}^*)^{\mathrm{t}}(F_{11} - F_{12}F_{22}^{-1}F_{21})\alpha _{(1)}^*\,\mathrm{d}s\Big \}^{-1}, \end{aligned}$$

as $K \rightarrow \infty$. The limit on the right, say $\Omega _{0,11}^{-1}$, is the expression of (4.3).

Suppose that there is a consistent estimator for $\theta _0$ with smaller variance than $\Omega _{0,11}^{-1}$ under the partly parametric partly nonparametric model in (1.4). Denote its variance matrix by *V*, so that $V < \Omega _{0,11}^{-1}$ (meaning that $V - \Omega _{0,11}^{-1}$ is a negative definite matrix). Since $\Omega _{K,11}^{-1} \le \Omega _{K+1,11}^{-1}$ for all *K* and $\Omega _{K,11}^{-1} \rightarrow \Omega _{0,11}^{-1}$ as $K \rightarrow \infty$, this means that there is a $K_0$ such that $V < \Omega _{K,11}^{-1}$ for all $K \ge K_0$. But $\Omega _{K,11}^{-1}$ is the Cramér–Rao lower bound for estimating $\theta _0$ under the *K*’th parametric model of the form (A.1), so $V < \Omega _{K,11}^{-1}$ cannot happen, and consequently there cannot be a consistent estimator for $\theta _0$ with smaller variance than $\Omega _{0,11}^{-1}$ under the model in (1.4).

## Efficiency and relative improvement calculations

There are general benefits from building and using parametric components models rather than nonparametric ones, provided the models can be assessed to check for adequacy, a theme addressed in the following section. In this section we consider questions related to efficiency; how much is gained, in precision, by using the parametric-nonparametric model (1.4), compared to the nonparametric Aalen methods?

### Asymptotic relative efficiencies

We have seen in previous sections that various limit distributions depend crucially on the limit matrix functions *F*, *G*, *H* of $F_n,G_n,H_n$, defined in Sect. 2, along with certain relatives. These functions will now be studied and compared for a certain setup, to illustrate also aspects of relative efficiency.

Assume that the censoring mechanism works independently of the life-times, with $\rho (s)=P\{C_i\ge s\}$ for its survival function. Then $\mathrm{E}\{Y_i(s)\,|\,z_i\}=\exp \{-z_i^\mathrm{t}A(s)\}\rho (s)$. With *Y*(*s*) and *Z* denoting generic at-risk indicator and covariate vector, distributed according the covariate distribution in question, we deduce

$$\begin{aligned} F(s)= & {} \mathrm{E}\, Y(s){ZZ^\mathrm{t}\over Z^\mathrm{t}\alpha (s)} =\mathrm{E}\exp \{-Z^\mathrm{t}A(s)\}{ZZ^\mathrm{t}\over Z^\mathrm{t}\alpha (s)}\,\rho (s) =F_0(s)\rho (s), \\ G(s)= & {} \mathrm{E}\, Y(s)ZZ^\mathrm{t}=\mathrm{E}\exp \{-Z^\mathrm{t}A(s)\}ZZ^\mathrm{t}\,\rho (s) =G_0(s)\rho (s), \\ \mathrm{d}H(s)= & {} \mathrm{E}\,\exp \{-Z^\mathrm{t}A(s)\}ZZ^\mathrm{t}Z^\mathrm{t}\alpha (s)\rho (s)\,\mathrm{d}s =\mathrm{d}H_0(s)\rho (s), \end{aligned}$$

cf. Assumptions 1. Assume now that the rates $\alpha _j$ are constant. Then

$$\begin{aligned} F_0(s)=\mathrm{E}\,\exp (-sZ^\mathrm{t}\alpha )ZZ^\mathrm{t}/(Z^\mathrm{t}\alpha ), \end{aligned}$$

with derivatives $F_{0,j,k}'(s)=-G_{0,j,k}(s)$, and the next derivative gives $\mathrm{d}H_{0,j,k}(s)$. Suppose further that the covariates $Z_1,\ldots ,Z_r$ are independent with Laplace transforms $L_j(u)=\mathrm{E}\,\exp (-uZ_j)=\exp \{-u\psi _j(u)\}$. Then

$$\begin{aligned} L_j'(u)= & {} -\mathrm{E}\, Z_j\exp (-uZ_j)=-L_j(u)\psi _j'(u), \\ L_j''(u)= & {} \mathrm{E}\, Z_j^2\exp (-uZ_j)=L_j(u)\{\psi _j'(u)^2-\psi _j''(u)\}, \end{aligned}$$

which leads to

$$\begin{aligned} G_{0,j,k}(s)= & {} \mathrm{E}\,\exp (-sZ^\mathrm{t}\alpha )Z_jZ_k\\= & {} \Bigl [\prod _{i=1}^r\exp \{-\psi _i(\alpha _is)\}\Bigr ] \{\psi _j'(\alpha _js)\psi _k'(\alpha _ks) -\delta _{j,k}\psi _j''(\alpha _js)\}, \end{aligned}$$

where $\delta _{j,k}$ equals 1 when $j = k$, and zero otherwise. These functions may now be studied and integrated numerically to give *F* functions, for different scenarios. We shall be content to illustrate this here for the case where the $Z_j$s have gamma distributions. Taking $Z_j$ to be gamma $(c_j,\gamma _j)$, with Laplace transform $\gamma _j^{c_j}/(\gamma _j+u)^{c_j}$, one finds $\psi _j(u)=c_j(\gamma _j+u)^{-1}$ and $\psi _j''(u)=-c_j(\gamma _j+u)^{-2}$, so that

$$\begin{aligned} G_{0,j,k}(s)=\Bigl (\prod _{i=1}^r{\gamma _i\over \gamma _i+\alpha _is}\Bigr ) \Bigl \{{c_j\over \gamma _j+\alpha _js}{c_k\over \gamma _k+\alpha _ks} +\delta _{j,k}{c_j\over (\gamma _j+\alpha _js)^2}\Bigr \}. \end{aligned}$$

With the further specialisation that $\alpha _j$s are equal to a common $\alpha$, and similarly that the $Z_j$s come from the same gamma $(c,\gamma )$ distribution, some work leads to

$$\begin{aligned} G_0(s)= & {} g(s)(c^{-1}I_r + e_re_r^\mathrm{t}), \quad \mathrm{where\ }g(s)={c^2\gamma ^{cr}\over (\gamma +\alpha s)^{cr+2}}, \\ F_0(s)= & {} f(s)(c^{-1}I_r + e_re_r^\mathrm{t}), \quad \mathrm{where\ }f(s)={c^2\gamma ^{cr}\over (cr+1)\alpha (\gamma +\alpha s)^{cr+1}}, \\ \mathrm{d}H_0(s)= & {} h(s)\,\mathrm{d}s\,(c^{-1}I_r + e_re_r^\mathrm{t}), \quad \mathrm{where\ }h(s)={c^2\gamma ^{cr}(cr+2)\alpha \over (\gamma +\alpha s)^{cr+3}}, \end{aligned}$$

where $e_r=(1,\ldots ,1)^\mathrm{t}$ of length *r* and $I_r$ is the identity matrix of size $r\times r$.

Inside this particular setup, with constant hazard rates and independent covariates, we may now answer various questions related to relative efficiency.

(i) How much is precision increased, for large *n*, by using the $\breve{A}$ estimator with estimated optimal weights $\widetilde{w}_i(s)$ instead of the simpler $\widetilde{A}$ estimator with plain weights $w_i(s)=1$ (see Sect. 2)? We find

$$\begin{aligned} F^{-1}={1\over f\rho }\Bigl (cI_r-{c^2\over 1+cr}e_re_r^\mathrm{t}\Bigr ) \quad \mathrm{and} \quad G^{-1}\,\mathrm{d}H\,G^{-1} ={h\over g^2\rho }\Bigl (cI_r-{c^2\over 1+cr}e_re_r^\mathrm{t}\Bigr ), \end{aligned}$$

the latter function being by inspection

$$\begin{aligned} \mathrm{a.r.e.}={cr+2\over cr+1}={\xi r+2/\gamma \over \xi r+1/\gamma } \end{aligned}$$

times bigger than the first, writing $\xi =c/\gamma$ for the mean of the $Z_j$s. The variance matrices for the limiting distributions of $A^*$ and $\widetilde{A}$ are the integrals of these functions, so the asymptotic relative efficiency ratio is equal to the same constant. The variance reduction may be small, when *c* or $\gamma$ (for fixed $\xi =c/\gamma$) is large, but can be as big as nearly 2, which happens for *c* small or $\gamma$ small.

(ii) How much better are the parametric estimators $\widehat{\theta }_jt$ of $A_j(t)$ than their best nonparametric counterparts, under model conditions of constant rates $\alpha _j(s)=\theta _j$ for $j=1,\ldots ,p$? The best nonparametric estimators $A^*_{(1)}(t)$ have variance

$$\begin{aligned} \mathrm{Var}_\mathrm{nonpm}=\int _0^t(F^{-1})_{11}\,\mathrm{d}s=\int _0^t{1\over f\rho }\,\mathrm{d}s\, \Bigl (cI_p-{c^2\over 1+cr}e_pe_p^\mathrm{t}\Bigr ). \end{aligned}$$

The limit variance matrix of $\sqrt{n}(\widehat{\theta }-\theta )$ is the inverse of $\Omega _0=\int _0^\tau Q^{-1}\,\mathrm{d}s$, by Sect. 4.1. For the best choice of weight functions, $Q=(F^{-1})_{11}$ with consequent $Q^{-1}=(F^{11})^{-1}$, leading to $\Omega _0$ being $\int _0^\tau f\rho \,\mathrm{d}s$ times $\{cI_p-c^2(1+cr)^{-1}e_pe_p\}^{-1}$. The limit variance matrix for the $\widehat{\theta }_jt$ estimators therefore becomes

$$\begin{aligned} \mathrm{Var}_\mathrm{pm}=t^2\Omega _0^{-1}={t^2\over \int _0^\tau f\rho \,\mathrm{d}s} \Bigl (cI_p-{c^2\over 1+cr}e_pe_p^\mathrm{t}\Bigr ). \end{aligned}$$

In order to reach more concrete comparisons, we let the censoring distribution be of the shifted Pareto type $\rho (s)=(1+\alpha s/\gamma )^{-k}$, with density $(k\alpha /\gamma )(1+\alpha s/\gamma )^{-(k+1)}$. We also let $\tau =\infty$. The distribution is stochastically increasing with decreasing *k*, with median equal to $(\gamma /\alpha )(2^{1/k}-1)$. The case of no censoring corresponds to $k=0$, while larger *k* corresponds to more heavy censoring. One finds

$$\begin{aligned} \int _0^\infty f\rho \,\mathrm{d}s= & {} {c^2\over \alpha ^2} {1\over (cr+1)(cr+k)} \mathrm{\ and\ } \int _0^t{1\over f\rho }\,\mathrm{d}s\\= & {} {cr+1\over cr+k+2}{\gamma ^2\over c^2} \Bigl \{\Bigl (1+{\alpha \over \gamma }t\Bigr )^{cr+k+2}-1\Bigr \}. \end{aligned}$$

The asymptotic inefficiency ratio becomes

$$\begin{aligned} {\mathrm{Var}_\mathrm{nonpm}\over \mathrm{Var}_\mathrm{pm}} ={1\over (cr+k)(cr+k+2)}{(1+u)^{cr+k+2}-1\over u^2}, \quad \mathrm{where\ }u=(\alpha /\gamma )t. \end{aligned}$$

Note that the two matrices are simply proportional to each other, and the ratio is independent of *p*.

(iii) How much improvement is there for the semiparametric estimators $\widehat{A}_{(2)}$ of $A_{p+1},\ldots ,A_{p+q}$, constructed in Sect. 4.2, compared to the nonparametric $A^*_{(2)}$? The latter ones have limit distribution variance matrix

$$\begin{aligned} \int _0^t(F^{-1})_{22}\,\mathrm{d}s=\int _0^t{1\over f\rho }\,\mathrm{d}s\, \Bigl (cI_q-{c^2\over 1+cr}e_qe_q^\mathrm{t}\Bigr ). \end{aligned}$$

This needs to be compared to the (4.5) formula. It involves $\phi (s)$, which in this situation is seen to simply be $F_{21}(s)$, so that

$$\begin{aligned} J(t)=\int _0^tF_{22}^{-1}F_{21}\,\mathrm{d}s =\int _0^t{1\over f\rho }f\rho \,\mathrm{d}s \Bigl (cI_q-{c^2\over 1+cq}e_qe_q^\mathrm{t}\Bigr )e_qe_p^\mathrm{t}=t{c\over 1+cq}e_qe_p^\mathrm{t}. \end{aligned}$$

The variance matrix formula (4.5) is found to be equal to

$$\begin{aligned} \int _0^t{1\over f\rho }\,\mathrm{d}s\Bigl (cI_q-{c^2\over 1+cq}e_qe_q^\mathrm{t}\Bigr ) +{t^2\over \int _0^\tau f\rho \,\mathrm{d}s} {c^3p\over (1+cq)(1+cr)}e_qe_q^\mathrm{t}. \end{aligned}$$

With the censoring mechanism given above, one finds the asymptotic inefficiency ratio, corresponding to the nonparametric limit variance of the $A_j$ estimator divided by the parametric limit variance, is

$$\begin{aligned} {1+c(r-1)\over 1-cr}v(u)\Big / \Bigl \{{1+c(q-1)\over 1-cq}v(u)+c^2\kappa (c,k)u^2\Bigr \}, \end{aligned}$$

where again $$u=(\alpha /\gamma )t$$,

$$\begin{aligned} v(u)=(1+u)^{cr+k+2}-1, \quad \mathrm{and} \quad \kappa (c,k)={(cr+k+2)p(cr+k)\over (cq+1)(cr+1)}. \end{aligned}$$

This ratio can now be studied, as a curve in *t*, for different sets of parameters. In certain setups the precision of the parametric estimator can be significantly better than the nonparametric one.

### Efficiency improvements for the plain-weights estimators

In the previous subsection we have cared for the particular case of theoretically optimal estimators, for $\widehat{\theta }$ and $\widehat{A}_{j+1},\ldots ,\widehat{A}_{p+q}$. These involve the use of certain cumbersome optimal weights $w_i(s)=1/z_i^\mathrm{t}\widetilde{\alpha }(s)$, an accompanying optimal $V_n(s)$ matrix when minimising the criterion function $C_n(\theta )$ of (3.1), and so on. This has led to relatively clear and transpararent formulae for relevant relative efficiency ratios.

In practice we would be more interested in similar efficiency ratios for our favoured default choice $V_n(s)=n^{-1}\sum _{i=1}^nY_i(s)z_iz_i^\mathrm{t}$, however. Propositions 4.1 and 4.2 may be used to find limit variance expressions in this case too, involving

$$\begin{aligned} n^{-1}\sum _{i=1}^nY_i(s)z_iz_i^\mathrm{t}&\rightarrow _p&\mathrm{E}\,\exp \{-Z^\mathrm{t}A(s)\}ZZ^\mathrm{t}\,\rho (s), \\ n^{-1}\sum _{i=1}^nY_i(s)z_iz_i^\mathrm{t}z_i^\mathrm{t}\alpha (s)&\rightarrow _p&\mathrm{E}\,\,\exp \{-Z^\mathrm{t}A(s)\}ZZ^\mathrm{t}\,Z^\mathrm{t}\alpha (s)\,\rho (s), \end{aligned}$$

and yet other quantities; again expectation is with respect to the ergodic distribution of covariates, see Assumptions 1. These expressions can be evaluated numerically, along with other required quantities, for given setups of covariance distributions and $A_j(t)$ functions. The efficiency ratios do not have clear formulae, however, so comparisons are harder and less transparent compared to those in the previous subsection. We have carried out some numerical computations, in simple cases, and found ratios broadly similar to those reached above.

### Improvement potential for a given problem

For a given dataset, and a given focus parameter $\mu =\mu (A_1,\ldots ,A_r)$, a statistician may compute two estimators: the $\widehat{\mu }_\mathrm{Aalen}=\mu (\widetilde{A}_1,\ldots ,\widetilde{A}_r)$ using (2.2) to estimate the cumulative regression functions, and

$$\begin{aligned} \widehat{\mu }_\mathrm{Partly} = \mu (A_1(\cdot ,\widehat{\theta }),\ldots ,A_p(\cdot ,\widehat{\theta }),\widehat{A}_{p+1}, \ldots ,\widehat{A}_{p+q}). \end{aligned}$$

Crucially, one may also use variance formulae developed in Sect. 4 to compute their standard errors, i.e. estimated standard deviations. For example, a focus parameter of particular interest in survival analysis is the survival function evaluated in some fixed time point *t*. For an individual with covariates $z_{0} = (z_{0,(1)}^{\mathrm{t}},z_{0,(2)}^{\mathrm{t}})^{\mathrm{t}}$, so that the focus parameter is $\mu = \exp \{-z_0^{\mathrm{t}}A(t)\}$, we have $\sqrt{n}(\widehat{\mu }_\mathrm{Partly} - \mu ) \rightarrow _d \mathrm{N}(0, \mu ^2 z_0^{\mathrm{t}}\Xi (t)z_0)$, where $\Xi (t)$ is the matrix appearing in (4.5).

A direct comparison of the standard errors of $\widehat{\mu }_\mathrm{Aalen}$ and $\widehat{\mu }_\mathrm{Partly}$ for a given focus parameter allows one to see if there is a clear gain in going from nonparametric to parametric for the $\alpha _j(s)$ components in question. This is illustrated in Fig. 3, with proof-of-the-pudding plots of the standard errors for the two estimators of the cumulatives $A_1(t),A_2(t),A_3(t)$, and also exemplified by the survival curve plot of Fig. 4 where the confidence band of the partly parametric survival curve is visibly more narrow than the confidence band corresponding to the Aalen estimator.
